# Ischemic stroke alters immune cell niche and chemokine profile in mice independent of spontaneous bacterial infection

**DOI:** 10.1002/iid3.277

**Published:** 2019-11-05

**Authors:** Breanne Y. Farris, Kelly L. Monaghan, Wen Zheng, Courtney D. Amend, Heng Hu, Amanda G. Ammer, James E. Coad, Xuefang Ren, Edwin C. K. Wan

**Affiliations:** ^1^ Department of Microbiology, Immunology, and Cell Biology West Virginia University School of Medicine Morgantown West Virginia; ^2^ Department of Physiology and Pharmacology West Virginia University School of Medicine Morgantown West Virginia; ^3^ Experimental Stroke Core, Center for Basic and Translational Stroke Research West Virginia University School of Medicine Morgantown West Virginia; ^4^ Pathology Laboratory for Translational Medicine West Virginia University School of Medicine Morgantown West Virginia; ^5^ Department of Neuroscience West Virginia University School of Medicine Morgantown West Virginia; ^6^ Rockefeller Neuroscience Institute West Virginia University School of Medicine Morgantown West Virginia

**Keywords:** chemokines, pulmonary immunity, stroke

## Abstract

**Introduction:**

Stroke‐associated pneumonia (SAP) is a major cause of mortality in patients who have suffered from severe ischemic stroke. Although multifactorial in nature, stroke‐induced immunosuppression plays a key role in the development of SAP. Previous studies using a murine model of transient middle cerebral artery occlusion (tMCAO) have shown that focal ischemic stroke induction results in functional defects of lymphocytes in the spleen, thymus, and peripheral blood, leading to spontaneous bacterial infection in the lungs without inoculation. However, how ischemic stroke alters immune cell niche and the expression of cytokines and chemokines in the lungs has not been fully characterized.

**Methods:**

Ischemic stroke was induced in mice by tMCAO. Immune cell profiles in the brain and the lungs at 24‐ and 72‐hour time points were compared by flow cytometric analysis. Cytokine and chemokine expression in the lungs were determined by multiplex bead arrays. Tissue damage and bacterial burden in the lungs following tMCAO were evaluated.

**Results:**

Ischemic stroke increases the percentage of alveolar macrophages, neutrophils, and CD11b^+^ dendritic cells, but reduces the percentage of CD4^+^ T cells, CD8^+^ T cells, B cells, natural killer cells, and eosinophils in the lungs. The alteration of immune cell niche in the lungs coincides with a significant reduction in the levels of multiple chemokines in the lungs, including CCL3, CCL4, CCL5, CCL17, CCL20, CCL22, CXCL5, CXCL9, and CXCL10. Spontaneous bacterial infection and tissue damage following tMCAO, however, were not observed.

**Conclusion:**

This is the first report to demonstrate a significant reduction of lymphocytes and multiple proinflammatory chemokines in the lungs following ischemic stroke in mice. These findings suggest that ischemic stroke directly impacts pulmonary immunity.

**Abbreviations**
BALF: bronchoalveolar lavage fluid
cDC: conventional dendritic cell
CNS: central nervous system
DC: dendritic cell
HPA: hypothalamic‐pituitary‐adrenal
moDC: monocyte‐derived dendritic cell
NK: natural killer
NKT: natural killer T
pDC: plasmacytoid dendritic cell
SAP: stroke‐associated pneumonia
SNS: sympathetic nervous system
tMCAO: transient middle cerebral artery occlusion

## 1 INTRODUCTION

Stroke is the fifth highest cause for overall mortality and the leading cause of long‐term disability in the United States.^1^ A major cause of mortality is the acquisition of stroke‐associated pneumonia (SAP). It was long thought that dysphagia‐mediated aspiration following stroke is the sole cause of SAP. However, it is now clear that the stroke‐induced immunosuppression is a critical risk factor for acquiring SAP,^2^ and although it has been recognized for more than 15 years,^3^ the cellular and molecular mechanisms that trigger this event are still not well‐defined. One possibility is that inflammatory immune cells infiltrating into the central nervous system (CNS) from the periphery during reoxygenation (reperfusion) causes transient exhaustion of immune cells in the periphery, leading to immunosuppression.^4^ Another explanation currently under active investigation is that brain damage in stroke triggers the hypothalamic‐pituitary‐adrenal axis and sympathetic nervous system in an attempt to dampen further inflammation within the CNS, causing bystander peripheral immunosuppression. These pathways stimulate the secretion of glucocorticoid and catecholamine respectively, both of which are natural immunosuppressive agents. The importance of neurological and endocrinal control of stroke‐induced immunosuppression was demonstrated in mouse models with the use of a β‐adrenoreceptor blocker propranolol to prevent poststroke pneumonia.^3^, ^5^ However, a recent historical cohort study concluded that the β‐blocker therapy does not reduce the risk of SAP in humans.^6^ Therefore, detailed investigation on how different immune cell types are affected quantitatively and functionally after stroke is critical for designing more specific therapeutic strategies.

Current knowledge of the immunological responses following stroke were mostly generated using the murine transient middle cerebral artery occlusion (tMCAO) model of ischemic stroke induction. So far, immune phenotypes that are linked to stroke‐induced immunosuppression include (a) induction of apoptosis of lymphocytes (T cells, B cells, and natural killer [NK] cells) in the blood, spleen, and the thymus^3^; (b) reduced expression of proinflammatory tumour necrosis factor (TNF) but increased expression of immunosuppressive interleukin‐10 (IL‐10) in blood and splenic monocytes^7^; (c) reduction of interferon γ (IFN‐γ) expression in blood T cells and liver natural killer T (NKT) cells^3^, ^8^; and (d) impaired neutrophil chemotaxis.^9^ Among these mechanisms, the reduction of IFN‐γ in liver NKT cells seems to be the most relevant, as propranolol treatment in mice following ischemic stroke restores IFN‐γ production in these cells.^8^ However, currently there is no explanation for how immune cell defects occurring in remote organs, such as the liver and the spleen, lead to bacterial infection in the lungs. Additionally, there is little information on how ischemic stroke affects the immune cell niche and functions in the lungs, which directly impact pulmonary immunity.

We predict that ischemic stroke alters lung immune cell quantity and/or function. The objectives of the study are (a) to investigate whether ischemic stroke induction in mice alters the immune cell niche in the lungs, and (b) to determine whether changes of the immune cell niche in the lungs following ischemic stroke induction result in spontaneous bacterial infection. Here, we report that the immune cell niche in the lungs is altered following the tMCAO‐mediated ischemic stroke induction, with a significant depletion of lymphocyte populations. Importantly, these changes do not result in spontaneous bacterial infections of the lungs and tissue damage as previously reported, but rather they correlate with the reduction of multiple chemokine levels in the lungs, which may represent immunosuppressive events following ischemic stroke.

## 2 MATERIALS AND METHODS

### 2.1 Animals

Eight to 12‐week‐old male C57BL/6J mice, weighing 25 to 30 g were used (The Jackson Laboratory, Bar Harbor, ME). The mice were housed under specific‐pathogen‐free conditions in the vivarium at West Virginia University Health Sciences Center. Mice were housed according to the Institutional Animal Care and Use Committee (IACUC) guidelines on a 12‐hour light/dark cycle and fed and watered ad libitum. All protocols and procedures performed were approved by the IACUC of West Virginia University (protocol number 1705006753).

### 2.2 Transient middle cerebral artery occlusion

tMCAO is the major animal model used to investigate the underlying mechanisms for the stroke‐induced immunological events. Mice were randomly assigned to either sham or tMCAO groups. tMCAO was performed at 8 to 11 am in the Experimental Stroke Core of West Virginia University. Mice were anesthetized with 4% to 5% isoflurane until a deep plane was reached and animals did not respond to toe‐pinch stimulus. Anesthesia was maintained using a nose cone during surgery with 1% to 2% isoflurane in oxygen enriched air. The common carotid artery and external carotid artery were exposed. Temporary suture was applied to the common carotid artery to stem the flow of blood for filament placement, and an incision was made in the external carotid artery. tMCAO was induced by inserting a monofilament into the external carotid artery which was further propelled to the middle cerebral artery, where it was left in place for 60 minutes. Following the 60‐minute period of ischemia, the filament was removed and reperfusion was allowed to occur. The sham operation included the suturing of the common carotid artery for 60 minutes without filament placement. Throughout surgical procedure and during reperfusion the flow of blood was monitored using Laser Doppler Flowmetery (Moor Instruments, UK). Mortality rate of the mice 72 hours following tMCAO was approximately 10%.

### 2.3 Neurological deficit assessment following tMCAO surgery

All mice were scored on a 6‐point neurological deficit scoring scale following sham or tMCAO procedure, and were reassessed every 24 hours for the duration of the study.^10^ 0—no neurological deficit, 1—retracts contralateral forepaw when lifted by the tail, 2—circles to the contralateral when lifted by the tail, 3—falling to the contralateral while walking, 4—does not walk spontaneously or is comatose, and 5—dead.

### 2.4 Triphenyltetrazolium chloride staining and quantification of infarct volume

Whole brains were removed from skull and surrounding tissue, then sectioned using a 2‐mm tissue matrix. Sections were stained in triphenyltetrazolium chloride at 37°C for 10 minutes on each side. Sections were then imaged, and total infarct volume was measured using Image J.

### 2.5 Brain tissue homogenization and single‐cell isolation

Brains were harvested 24 and 72 hours following tMCAO or sham operation, and were mechanically homogenized using a razor blade. Brain homogenates were digested with collagenase D (1 mg/mL) and DNase I (200 µg/mL) for 30 minutes at 37°C. Tissues were then passed through a 100‐µm cell strainer. Single cells were isolated by discontinuous Percoll gradient centrifugation.

### 2.6 Lung perfusion and excision

Mice were deeply anesthetized using ketamine/xylazine combination. Once in a deep plane of anesthesia, in which mice did not respond to toe‐pinch stimulus, thoracic cavity was exposed and whole‐body perfusion was performed by administering 15 mL of cold phosphate‐buffered saline (PBS) via the left cardiac ventricle. Lungs were excised and place into 2 mL of cold PBS supplemented with 1% fetal bovine serum (FBS). Right apical lobes were weighed for homogenization.

### 2.7 Lung tissue digestion and single‐cell isolation

Right cardiac, diaphragmatic, azygous, and left lobes were place into digestion buffer containing collagenase D (1 mg/mL) and DNase I (200 µg/mL) in Hank's Buffered Salt Solution. Lung lobes were inflated with 1 mL of digestion buffer and incubated at room temperature for 5 minutes. Following incubation, lung lobes were cut into pieces 2 to 3 mm in size and placed into 5 mL of digestion buffer. The tissues were vortexed and incubated in a 37°C water bath for 45 minutes with vigorous vortexing every 8 to 10 minutes. Digests were pushed through 100‐µm cell strainers and contents were centrifuged for 10 minutes at 380*g*. Supernatant was discarded and tissue digests were resuspended in PBS with 1% FBS to obtain single cell suspension.

### 2.8 Lung tissue homogenization

Right apical lobes were placed into a 2‐mL screw cap microtube containing (three) sterile 2.3 mm zirconia/silica microbeads (BioSpec Products, Bartlesville, OK) and 200 µL of cold PBS containing 1× HALT protease inhibitor cocktail (Thermo Fisher Scientific, Waltham, MA). Tissues were homogenized using the BeadBug™ benchtop microtube homogenizer on maximum speed for 2 minutes. Following homogenization, samples were transferred to prechilled tubes and centrifuged at 15 870*g* for 3 minutes. Supernatants were stored at −80°C for multiplex bead array analysis.

### 2.9 Lung tissue homogenization and culture for the assessment of spontaneous pneumonia

Mice were euthanized 24 and 72 hours following sham or tMCAO operation. Whole lungs were excised, rinsed in sterile PBS, and then mechanically homogenized in 1 mL of sterile PBS in a 7‐mL glass dounce tissue grinder (Corning, Corning, NY). Tissue homogenates were passed through a 100‐µm sterile cell strainer and serially diluted. Aliquots of serial dilution were plated onto Luria agar and incubated at 37°C overnight to assess for bacterial growth.

### 2.10 Lung tissue histopathology for the assessment of pneumonia

Mice were euthanized 24 and 72 hours following sham or tMCAO operation. Mice were tracheally cannulated and lungs were excised. Lungs were then inflated with 10% formalin. Tissue was fixed in formalin for a minimum of 24 hours before being embedded into paraffin, sectioned, and mounted onto the slides. Sections were stained with hematoxylin and eosin stain and assessed by a pathologist for the presence of histopathological features of pneumonia.

### 2.11 Immunohistochemistry for the assessment of activated caspase‐3

Mice were euthanized 72 hours following sham and tMCAO operation. Lung and spleen tissues were harvested, then fixed in 4% paraformaldehyde at 4°C overnight. After fixation, the tissues were embedded in tissue freezing medium, and sectioned to a thickness of 20 µm using cryostat. After 10 minutes incubation in 3% H_2_O_2_ (in methanol) at room temperature, the sections were incubated in the Tris‐buffered saline containing 0.3% Triton X 100% and 5% normal goat serum for 1 hour at room temperature, then incubated with primary antibody that recognizes the cleaved (Asp175) form of caspase 3 in a dilution of 1:500 (clone 5A1E, Cell Signaling Technology, Danvers, MA) overnight at 4°C. The sections were washed, then incubated with the SignalStain Boost IHC detection reagents (Cell Signaling Technology) for 30 minutes at room temperature. The horseradish peroxidase activity was detected with SignalStain DAB substrate kit (Cell Signaling Technology). The sections were counterstained with hematoxylin, dehydrated, and mounted. Images were collected with an Olympus Slide Scanner at 10x magnification.

### 2.12 Broncho‐alveolar lavage of the lungs

Mice were euthanized and tracheas were exposed. A cannula was inserted by a small incision into the trachea and secured with surgical suture. Thoracotomy was performed to expose lung tissue. Two fractions of a total of 3 mL cold PBS were instilled into the lungs: the first fraction of 0.4 mL was delivered, and then withdrawn following 30 seconds of continuous gentle lung massage. The second fraction of 2.6 mL were delivered in aliquots of 0.6‐0.7 mL. The aliquots were delivered and withdrawn with simultaneous and continuous gentle massage of the lungs. The first fraction was centrifuged at 470*g* for 5 minutes, and supernatant was stored at −80°C for multiplex bead array analysis. The second fraction was centrifuged at 470*g* for 5 minutes, and supernatant was discarded. The cell pellets from both fractions were combined in 1 mL of cold RPMI, quantified, and analyzed by flow cytometry.

### 2.13 Cell quantification and phenotyping by flow cytometry

Lung and brain single cell suspensions were quantified by the trypan blue exclusion method. Cells were blocked with anti‐CD16/32 (Biolegend, San Diego, CA); immune cell types were identified using combinations of antibody listed in Table 1. All antibodies were purchased from Biolegend, except anti‐Siglec F, which was purchased from BD Pharmingen (Franklin Lakes, NJ). LIVE/DEAD Fixable Dead Cell Stain was used to exclude dead cells (Thermo Fisher Scientific). Samples were run on a LSRFortessa (BD Biosciences) using FACSDiva software version 8.0, and analyzed using FlowJo version 9.9.6.

**Table 1:** Surface markers and antibody combinations for determining immune cells from the lungs and the brain following tMCAO

Antibody	Clone	Immune cell type	Population	Surface marker expression
CD45‐FITC	30‐F11	Alveolar macrophages	L1	CD45+ Siglec F+ CD11b−
Siglec F‐PE	E50‐2440	Eosinophils	L2	CD45+ Siglec F+ CD11b+
CD11c‐Percp/Cy5.5	N418	CD103+ DCs	L3	CD45+ Siglec F− CD11b− CD103+ CD11c+ MHC II+
CD11b‐PE/Cy7	M1/70	CD11b+ DCs	L4	CD45+ Siglec F− CD11b hi CD103− CD64− MHC II+
CD64‐APC	X54‐5/7.1	Interstitial macrophages	L5	CD45+ Siglec F− CD11b hi CD103− CD64+ MHC II+
Live/dead‐APC/Cy7				
CD103‐BV421	2E7			
MHC II‐BV510	M5/114.15.2			
CD45‐FITC	30‐F11	Monocytes/moDCs	L8	CD45+ CD11b hi Ly6C hi/int CCR2+/− Ly6G−
Ly6C‐PE	HK1.4	Neutrophils	L9	CD45+ CD11b hi Ly6C int CCR2− Ly6G+
CD11b‐PE/Cy7	M1/70			
Live/dead‐APC/Cy7				
CCR2‐BV421	SA203G11			
Ly6G‐BV510	1A8			
CD45‐FITC	30‐F11	Plasmacytoid DCs	L6	CD45+ B220+ CD11c+
CD8‐PE	53‐6.7	B cells	L7	CD45+ B220+ CD11c−
NK1.1‐Percp/Cy5.5	PK136	CD4+ T cells	L10	CD45+ B220− CD11c− CD4+ CD8−
CD11c‐PE/Cy7	N418	CD8+ T cells	L11	CD45+ B220− CD11c− CD4− CD8+
B220‐APC	RA3‐6B2	NK cells	L12	CD45+ B220− CD11c− CD4− CD8− NK1.1+ TCRβ−
Live/dead‐APC/Cy7		NKT cells	L13	CD45+ B220− CD11c− CD4− CD8− NK1.1+ TCRβ+
CD4‐BV421	GK1.5			
TCRβ‐BV510	H57‐597			

Abbreviations: FITC, fluorescein isothiocyanate; moDC, monocyte‐derived dendritic cell; NK, natural killer; NKT, natural killer T; tMCAO,transient middle cerebral artery occlusion.

### 2.14 Multiplex cytokine and chemokine bead arrays

Multiplex bead arrays for cytokine and chemokine quantification were performed according to manufacturer protocol (catalogue #740446 and #740451; Biolegend). Samples were analyzed on a LSRFortessa (BD Biosciences) as described above.

### 2.15 Statistical analyses

Statistical comparison between samples was done by Student's *t* test using either GraphPad Prism 7, or an online tool developed by the College of Saint Benedict and Saint John's University, Collegeville, MN (http://www.physics.csbsju.edu/stats/t‐test.html; *P < .05; **P < .01; ***P < .001; not statistically different [NS]).

## 3 RESULTS

### 3.1 Severe ischemic stroke in C57BL/6J mice does not cause spontaneous pneumonia

**Figure 1:**
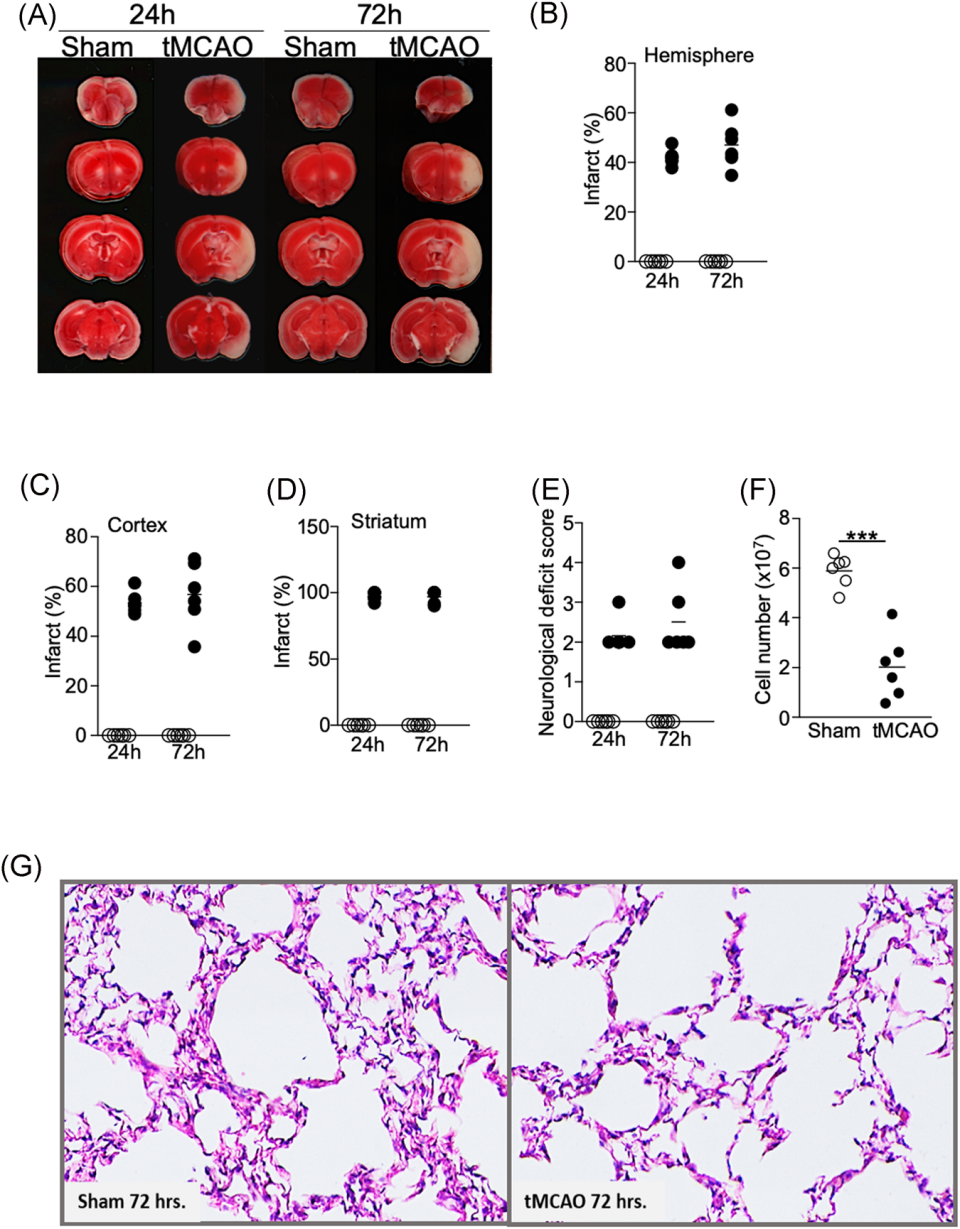
Severe ischemic stroke in C57BL/6J mice does not cause spontaneous pneumonia. Brain, spleen, and lung tissues were analyzed 24 and 72 hours following tMCAO or sham operation. A, Representative images showing ipsilateral brain infarcts following tMCAO but not sham operation by TTC staining. B‐D, Percentage of infarcts within the ipsilateral hemisphere (B), cortex (C), and corpus striatum (D) following tMCAO (filled circle) or sham controls (open circle) quantified by Image J. E, Neurological deficit scores of the mice 24 and 72 hours following tMCAO (filled circle) or sham controls (open circle). See Section 2 for score definition. F, Cell number from the spleens of mice 72 hours following tMCAO (filled circle) or sham controls (open circle). G, Representative images from H&E staining of lung tissues 72 hours following tMCAO (right) or sham operation (left). Images from all animals are shown in Figure S1. Data shown are combined results from two independent experiments with *n* = 6 animals per group (sham 24 hours, tMCAO 24 hours, sham 72 hours, and tMCAO 72 hours). ***P < .001. H&E, hematoxylin and eosin; tMCAO, transient middle cerebral artery occlusion; TTC, Triphenyltetrazolium chloride

**Figure 2:**
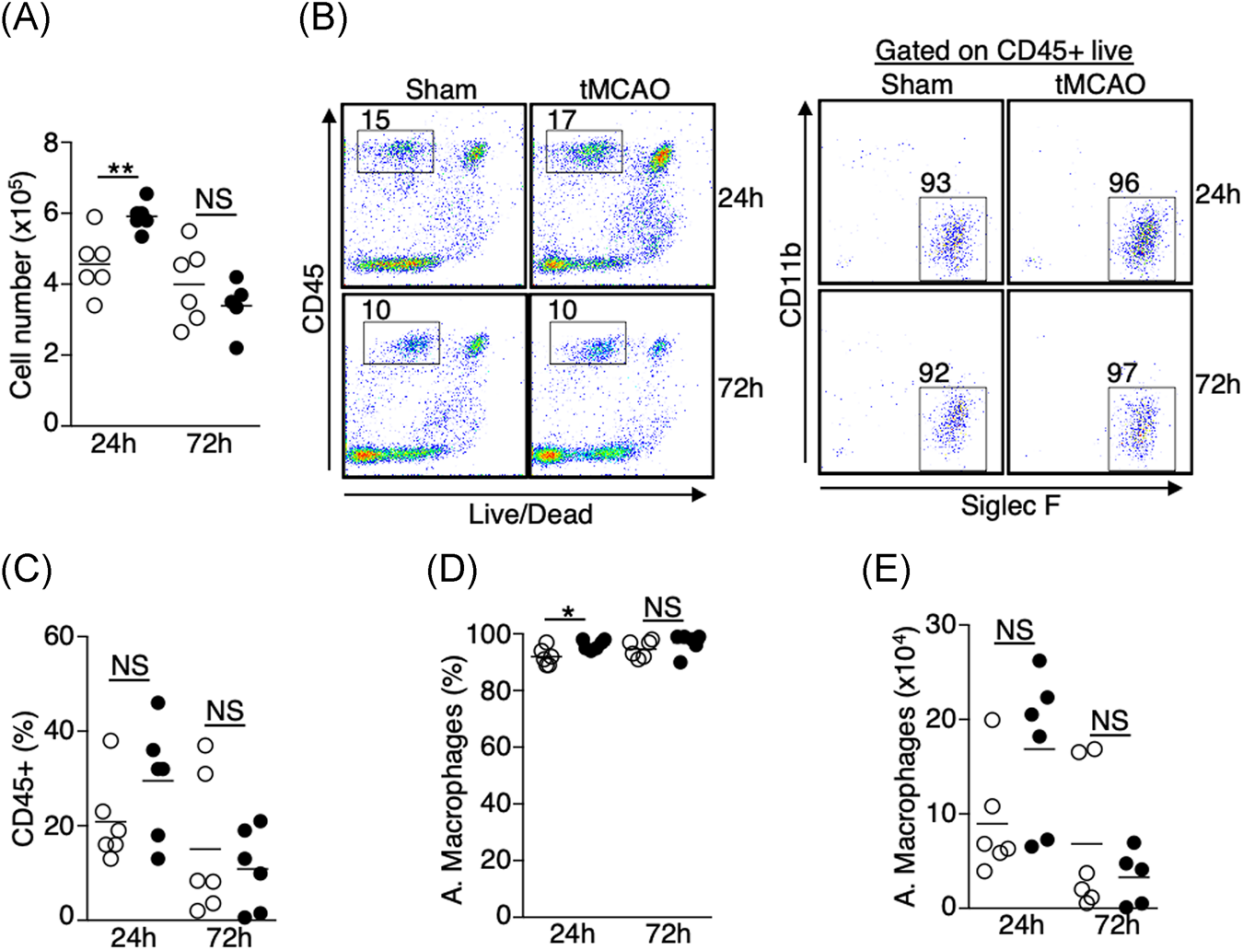
Increase in the number of alveolar macrophages in the BALF 24 hours postischemic stroke. A, Total number of cells recovered from BALF 24 and 72 hours following tMCAO (filled circle) or sham operation (open circle). B, Cellular compositions of BALF 24 and 72 hours following tMCAO. B, Representative plots showing percentage of CD45+ cells (left) and alveolar macrophages (right), which are defined as CD45+ Siglec F+ CD11b−. C‐E, Graphs showing percentage of CD45+ cells (C); percentage (D) and number (E) of alveolar macrophages of individual animals described in (B). tMCAO (filled circle) and sham operation (open circle). Data shown are combined results from two independent experiments with *n* = 6 animals per group (sham 24 hours; tMCAO 24 hours; sham 72 hours; tMCAO 72 hours). *P < .05; **P < .01. BALF, bronchoalveolar lavage fluid; NS, not statistically different; tMCAO, transient middle cerebral artery occlusion

Several groups reported that ischemic stroke induced by tMCAO in mice causes spontaneous pneumonia, defined as lung infection without bacterial inoculation. However, previous studies have shown that the incidence of spontaneous pneumonia varies in mice with different genetic backgrounds,^11^ and may also depend on environmental factors such as animal housing facility conditions.^12^, ^13^ We performed tMCAO in C57BL/6J mice by inserting a monofilament into the middle cerebral artery for 60 minutes, followed by monofilament removal to allow blood reperfusion. We obtained significant brain infarcts at 24 and 72 hours following tMCAO (Figure 1A,B). The percentage of infarcts in the ipsilateral cortex was over 50% and nearly 100% in the corpus striatum (Figure 1C,D). Correspondingly, neurological deficits of score greater than or equal to 2 were observed (Figure 1E). In addition, we found a significant reduction in splenic cellularity (Figure 1F) in mice following tMCAO, which is a feature of severe ischemic stroke in humans and mice.^3^, ^14^ These data indicate that induction of severe ischemic stroke was achieved. However, we could not detect spontaneous pneumonia in our mice following tMCAO. Pathological characteristics of bacterial pneumonia, such as inflammation, tissues damage, and edema were not observed in the lungs (Figures 1G and S1). The cellularity of the bronchoalveolar lavage fluid (BALF) was modestly increased in mice 24 hours following tMCAO compared to sham operation (Figure 2A). Over 90% of CD45+ cells were alveolar macrophages (Figure 2B‐E), but not neutrophils, which are commonly found in BAL during bacterial pneumonia. There was no bacterial recovery in lung homogenates 24 hours following tMCAO (Table S1). At 72 hours, a low quantity of bacteria were detected in some tMCAO‐ and sham‐operated mice (Table S1), but the quantity was substantially lower than that which has previously been reported.^3^, ^15^ Our findings suggest that there are factors other than the severity of the stroke that determine the incidence of spontaneous pneumonia in mice. In our studies, bacterial infection without inoculation was either absent or below detectable levels.

### 3.2 Alterations of the resident innate immune cell niche in the lungs following ischemic stroke

**Figure 3:**
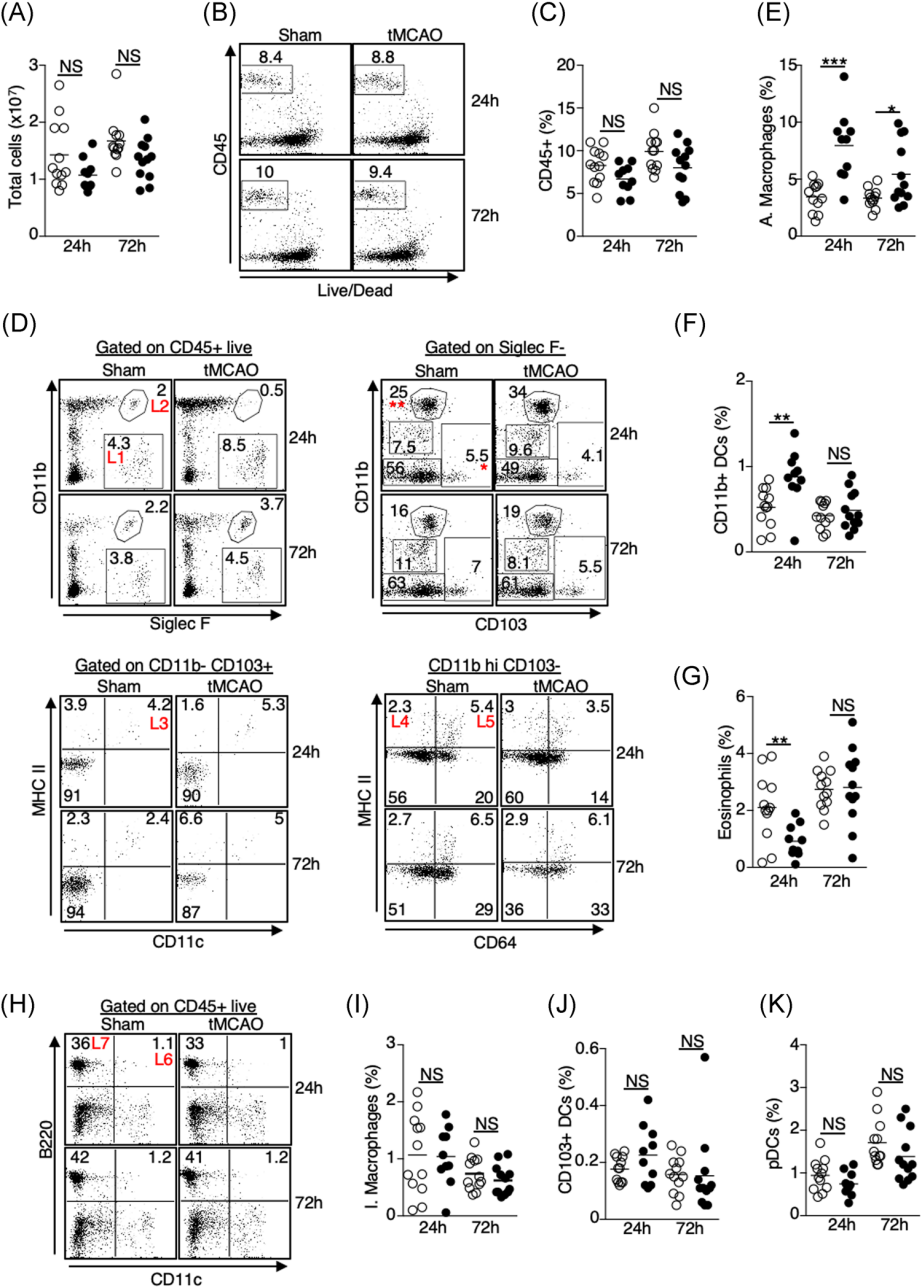
Alterations in the resident innate immune cell niche in the lungs following ischemic stroke. Lung tissues were excised 24 and 72 hours following tMCAO (filled circle) or sham operation (open circle), resident innate immune cells in the lungs were analyzed by flow cytometry, defined by surface markers listed on Table 1. A, Graph showing total number of cells in the lungs of individual animals. B, Representative plots showing percentage of CD45+ cells. C, Graph showing percentage of CD45+ cells in the lungs of individual animals. D, Representative plots showing the identification of alveolar macrophages (L1), eosinophils (L2), CD103+ DCs (L3), CD11b+ DCs (L4), and interstitial macrophages (L5). CD103+ DCs were first gated on CD103+ CD11b− cells (*). CD11b+ DCs and interstitial macrophages were first gated on CD11b hi CD103− cells (**). E‐G, Graphs showing percentage of alveolar macrophages (E), CD11b+ DCs (F), and eosinophils (G) of individual animals. H, Representative plots showing that identification of pDCs (L6), and B cells (L7, discussed in Figure 5). I‐K, Graphs showing percentage of interstitial macrophages (I), CD103+ DCs (J), and pDCs (K) of individual animals. Data shown are combined results from three independent experiments with *n* = 12 animals per group (sham 24 hours; tMCAO 24 hours; sham 72 hours; tMCAO 72 hours). **P < .01; ***P < .001. NS, not statistically different; pDC, plasmacytoid dendritic cell; tMCAO, transient middle cerebral artery occlusion

Resident innate immune cells such as macrophages and dendritic cells (DCs) are the first line of defense during pulmonary bacterial infection. We first determined the quantitative changes of lung‐resident alveolar and interstitial macrophages, CD11b+ and CD103+ conventional dendritic cells, plasmacytoid dendritic cells (pDCs), and eosinophils 24 and 72 hours following tMCAO by flow cytometry (Table 1). The total number of cells and the percentage of CD45+ cells in the lungs were slightly lower but did not reach statistical significance (Figure 3A‐C). Given that some of these innate immune cells only contribute to less than 0.1% of the total lung cells, data is presented as percent change within the CD45+ population. Twenty‐four hours following ischemic stroke, we found a significant increase of alveolar macrophages and CD11b+ DCs in the lungs, whereas eosinophils were reduced (Figure 3D‐G). The increase of alveolar macrophages persisted up to 72 hours following ischemic stroke, albeit to a lesser extent (Figure 3D,E). However, at 72 hours the number of CD11b+ DCs and eosinophils were comparable to mice with sham‐operation (Figures 3D and 3F). The percentages of interstitial macrophages, CD103+ DCs, and pDCs were unchanged at both time points (Figure 3D,H‐K). These data suggest that ischemic stroke alters specific subsets of immune cells in the pulmonary environment, particularly 24 hours poststroke.

### 3.3 Increased infiltration of neutrophils but not monocytes to the lungs following ischemic stroke despite an elevation of CCL2

**Figure 4:**
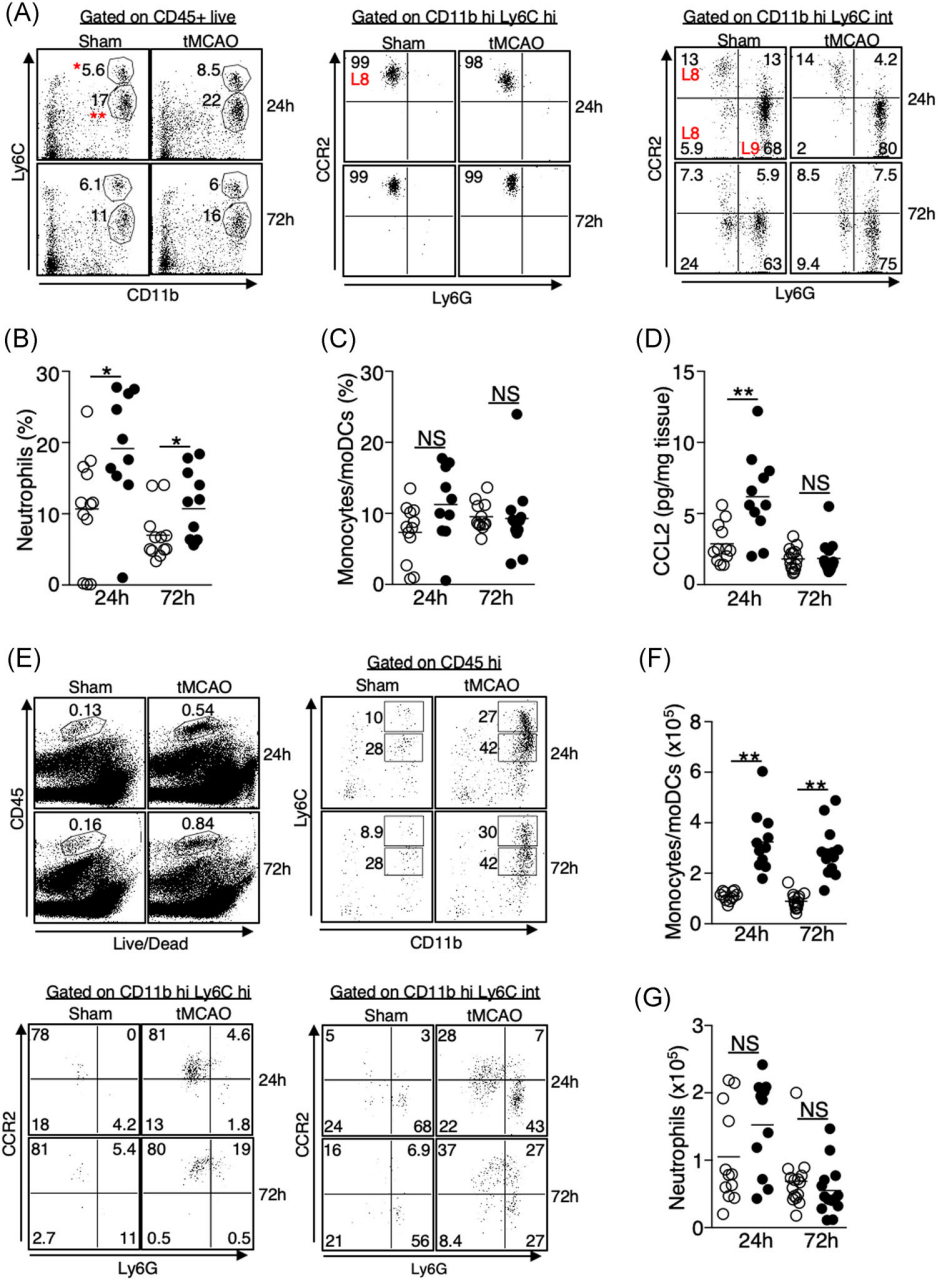
Increased infiltration of neutrophils but not monocytes to the lungs following ischemic stroke despite an elevation of CCL2. A‐C, Lung tissues were excised 24 and 72 hours following tMCAO or sham operation. Monocytes/moDCs and neutrophils in the lungs were analyzed by flow cytometry, defined by surface markers listed on Table 1. A, Representative plots showing the identification of monocytes/moDCs (L8) and neutrophils (L9) in the lungs. Monocytes/moDCs were defined as CD45+ Ly6C hi (*) CCR2 hi (middle) and Ly6C intermediate (**) CCR2+/− (right). Neutrophils were defined as CD45+ Ly6C intermediate (**) Ly6G+ (right). B‐C, Graphs showing percentage of neutrophils (B) and monocytes/moDCs (C) of individual animals 24 and 72 hours following tMCAO (filled circle) or sham operation (open circle). D, Lung tissues were homogenized 24 and 72 hours following tMCAO (filled circle) or sham operation (open circle), level of CCL2 was determined by multiplex bead array. E‐G, Brain tissues were excised 24 and 72 hours following tMCAO or sham operation, monocytes/moDCs and neutrophils in the brains were analyzed by flow cytometry. E, Representative plots showing the identification of monocytes/moDCs and neutrophils in the brains, which were defined as in (A). F,G, Graphs showing number of monocytes/moDCs (F) and neutrophils (G) of individual animals 24 and 72 hours following tMCAO (filled circle) or sham operation (open circle). Data shown are combined results from three independent experiments with *n* = 12 animals per group (sham 24 hours; tMCAO 24 hours; sham 72 hours; tMCAO 72 hours). *P < .05; **P < .01. moDC, monocyte‐derived dendritic cell; NS, not statistically different; tMCAO, transient middle cerebral artery occlusion

In addition to the activation of resident innate immune cells, neutrophil, and monocyte infiltration to the lungs plays a critical role in bacterial clearance.^16^, ^17^, ^18^ We determined whether ischemic stroke affects trafficking of these cells to the lungs. Flow cytometric analysis revealed an increase of neutrophils in the lungs 24 and 72 hours following tMCAO (Figure 4A,B), whereas the percentage of monocytes and monocyte‐derived dendritic cells (moDCs) was not changed (Figures 4A and 4C), despite an increase in the monocyte chemoattractant CCL2 (Figure 4D). Interestingly, there was a massive infiltration of monocytes and moDCs to the brain following ischemic stroke (Figure 4E,F), however, the number of neutrophils in the brain was unchanged (Figures 4E and 4G). These data suggest that ischemic stroke differentially directs monocyte and neutrophil trafficking into the brain and lungs, respectively.

### 3.4 Ischemic stroke leads to a significant loss of lymphocytes in the lungs independent of apoptosis

**Figure 5:**
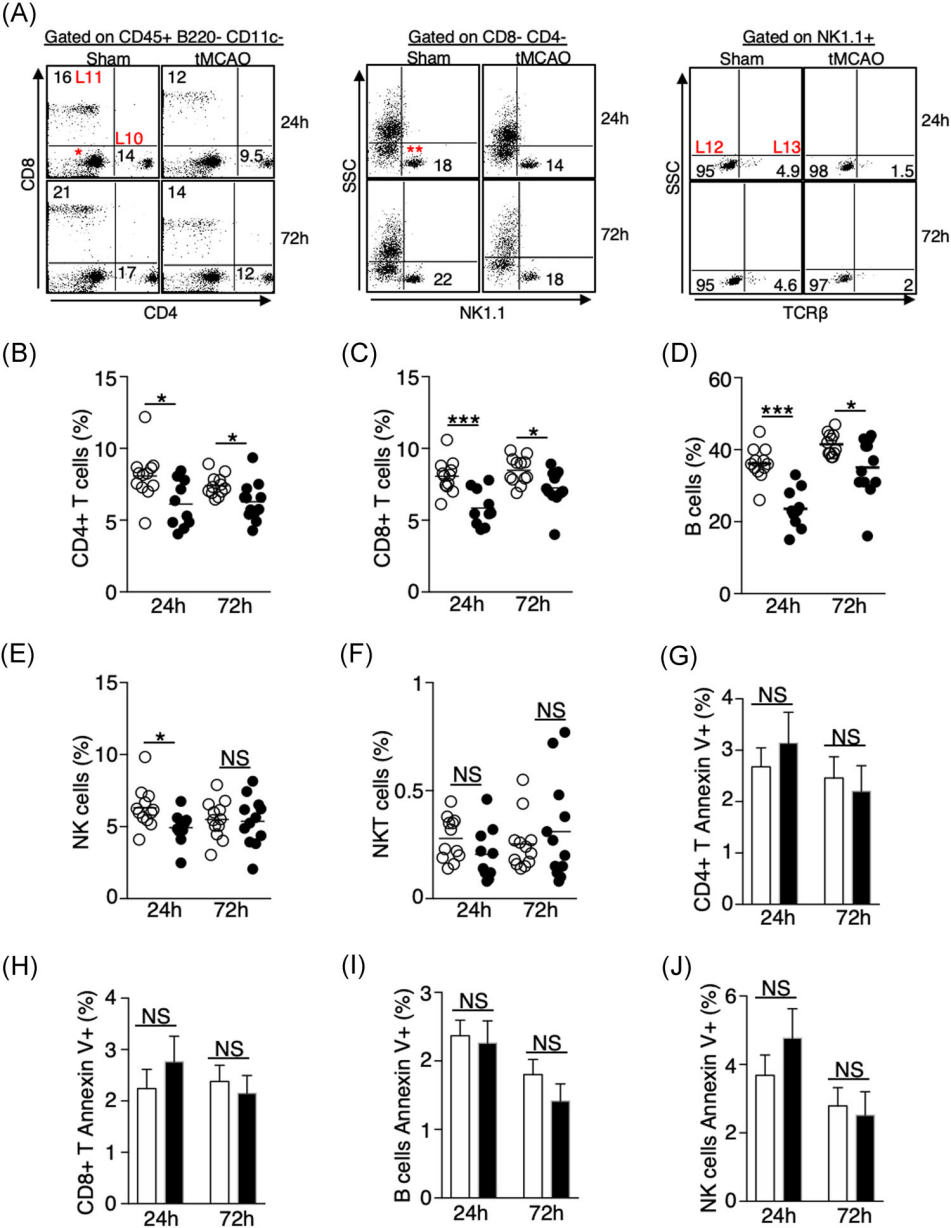
Ischemic stroke leads to a significant loss of lymphocytes in the lungs independent of apoptosis. A, Representative plots showing the identification of lymphocytes in the lungs by surface markers listed on Table 1. CD4+ (L10) and CD8+ (L11) T cells were first gated on CD45+ B220− CD11c− cells shown in Figure 3H. NK cells (L12) and NKT cells (L13) were gated on CD4− CD8− cells (*), then further gated on NK1.1+ cells (**). Representative plots for the identification of B cells shown in Figure 3H. B‐F, Graphs showing percentage of CD4+ T cells (B), CD8+ T cells (C), B cells (D), NK cells (E), and NKT cells (F) of individual animals 24 and 72 hours following tMCAO (filled circle) or sham operation (open circle). G‐H, Graphs showing percentage of annexin‐V+ CD4+ T cells (G), CD8+ T cells (H), B cells (I), and NK cells (J) 24 and 72 hours following tMCAO (filled bar) or sham operation (open bar). Data shown are combined results from three independent experiments with *n* = 12 animals per group (sham 24 hours, tMCAO 24 hours, sham 72 hours, tMCAO 72 hours). *P < .05; ***P < .001. NKT, natural killer T; NS, not statistically different; tMCAO, transient middle cerebral artery occlusion

**Figure 6:**
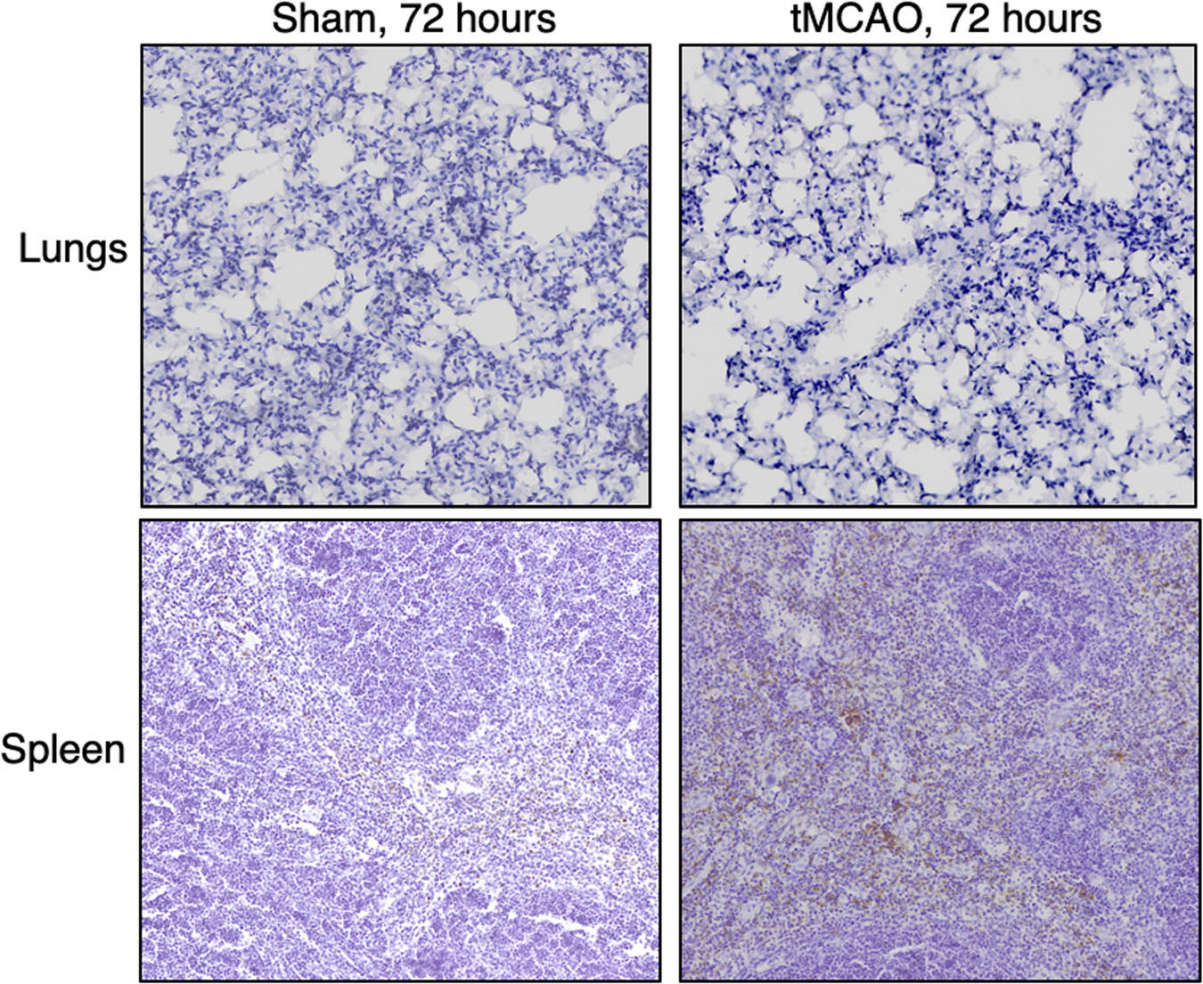
Ischemic stroke does not induce the activation of caspase 3 in the lungs. Spleen and lung tissues were dissected 72 hours following tMCAO or sham operation. The cleaved (activated) form of caspase 3 was measured by immunohistochemistry assay. Shown are representative images with *n* = 6 per group for the lung tissues and *n* = 3 per group for the spleens. Spleen samples following tMCAO serve as positive control. Brown color indicates positive signal. The tissues were counterstained with hematoxylin. Lung images from all animals are shown in Figure S2. tMCAO, transient middle cerebral artery occlusion

**Figure 7:**
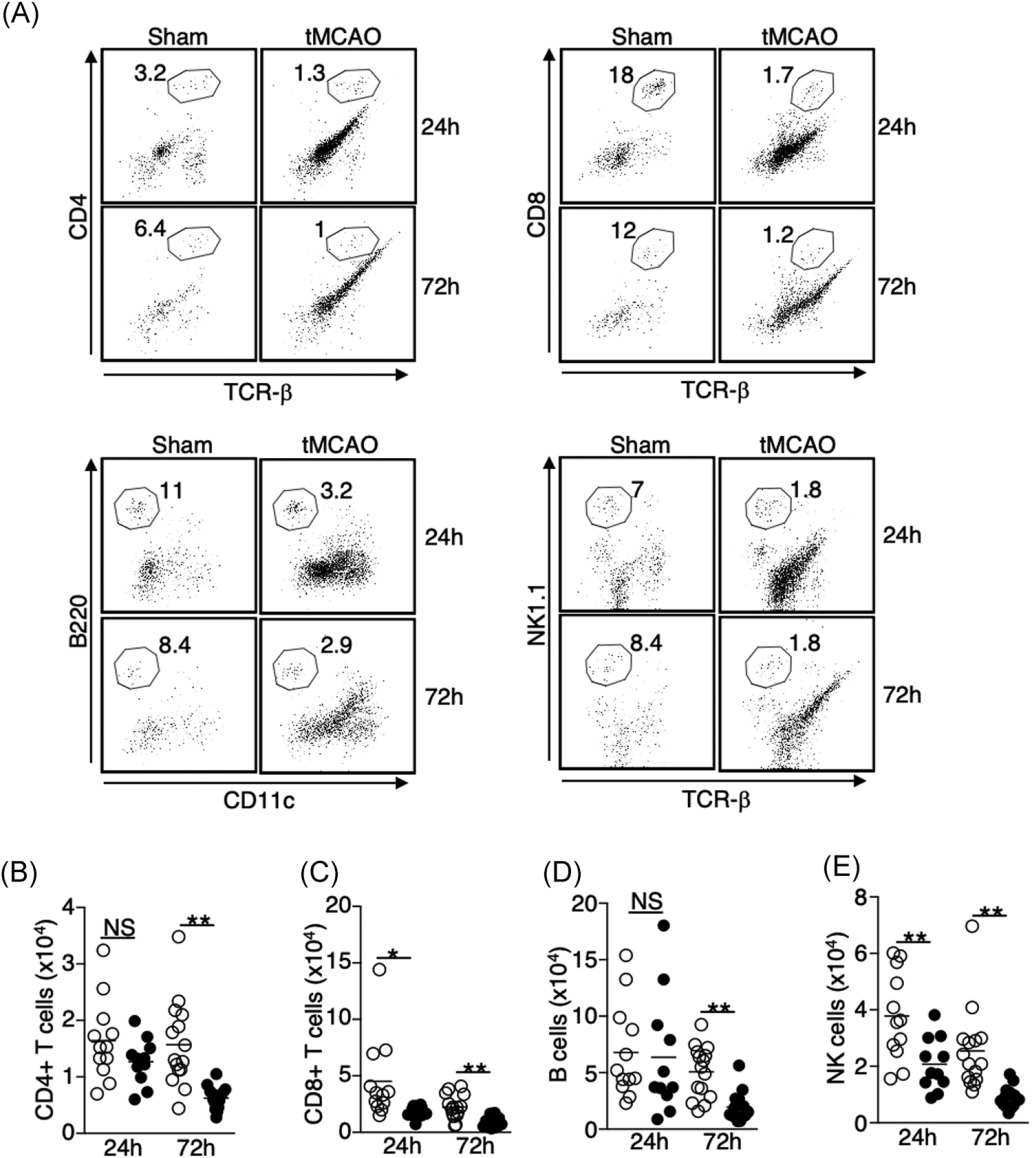
Loss of lymphocytes in the lungs following tMCAO is not the result of cell migration to the brain. A, Representative plots showing the identification of CD4+ T cells (CD4+ TCR‐β+, top left); CD8+ T cells (CD8+ TCR‐β+, top right); B cells (B220+ CD11c−, bottom left); and NK cells (NK1.1+ TCR‐β−, bottom right) in the brains. Cells were first gated on CD45 hi cells shown in Figure 4E. B‐E, Graph showing number of CD4+ T cells (B), CD8+ T cells (C), B cells (D), and NK cells (E) of individual animals 24 and 72 hours following tMCAO (filled circle) or sham operation (open circle). Data shown are combined results from three independent experiments with *n* = 12 animals per group (sham 24 hours, tMCAO 24 hours, sham 72 hours, tMCAO 72 hours). *P < .05; **P < .01. NS, not statistically different. TCR, T‐cell receptor; tMCAO, transient middle cerebral artery occlusion

Previous studies have shown that ischemic stroke leads to the apoptosis of T cells, B cells, and NK cells in the spleen, blood, and thymus.^3^ However, how ischemic stroke alters the lymphocyte population in the lungs is not clear. We found that the percentage of CD4+ T cells, CD8+ T cells, and B cells were significantly reduced 24 and 72 hours following tMCAO (Figures 3H and 5A‐D). NK cells were reduced at 24 but not 72 hours following tMCAO (Figures 5A and 5E), whereas NKT cells were unaltered in both time points (Figures 5A and 5F). We speculated that the loss of lymphocytes in the lungs was caused by increased apoptosis as was previously described in other tissues. We determined the percentage of apoptotic cells by annexin V staining. The percentage of apoptotic CD4+ T cells, CD8+ T cells, B cells, and NK cells were comparable in sham‐operated and tMCAO‐induced mice (Figure 5G‐J), suggesting that unlike other tissues, ischemic stroke leads to a significant loss of lymphocytes in the lungs but it is unrelated to the induction of apoptosis. Correspondingly, activation of caspase‐3 was not detected in the lung tissues following tMCAO by immunohistochemistry, but it was detected in the spleens, as previously described^3^ (Figures 6 and S2). We then investigated whether the reduction of lymphocytes in the lungs was caused by cell trafficking to the brain after ischemic stroke. Surprisingly, the number of CD4+ T cells, CD8+ T cells, B cells, and NK cells in the brain was either unchanged or reduced poststroke, indicating that the loss of lymphocytes in the lungs following tMCAO likely does not the result from cell infiltration to the brain (Figure 7A‐E).

### 3.5 Ischemic stroke suppresses the production of multiple chemokines and cytokines in the lungs

**Figure 8:**
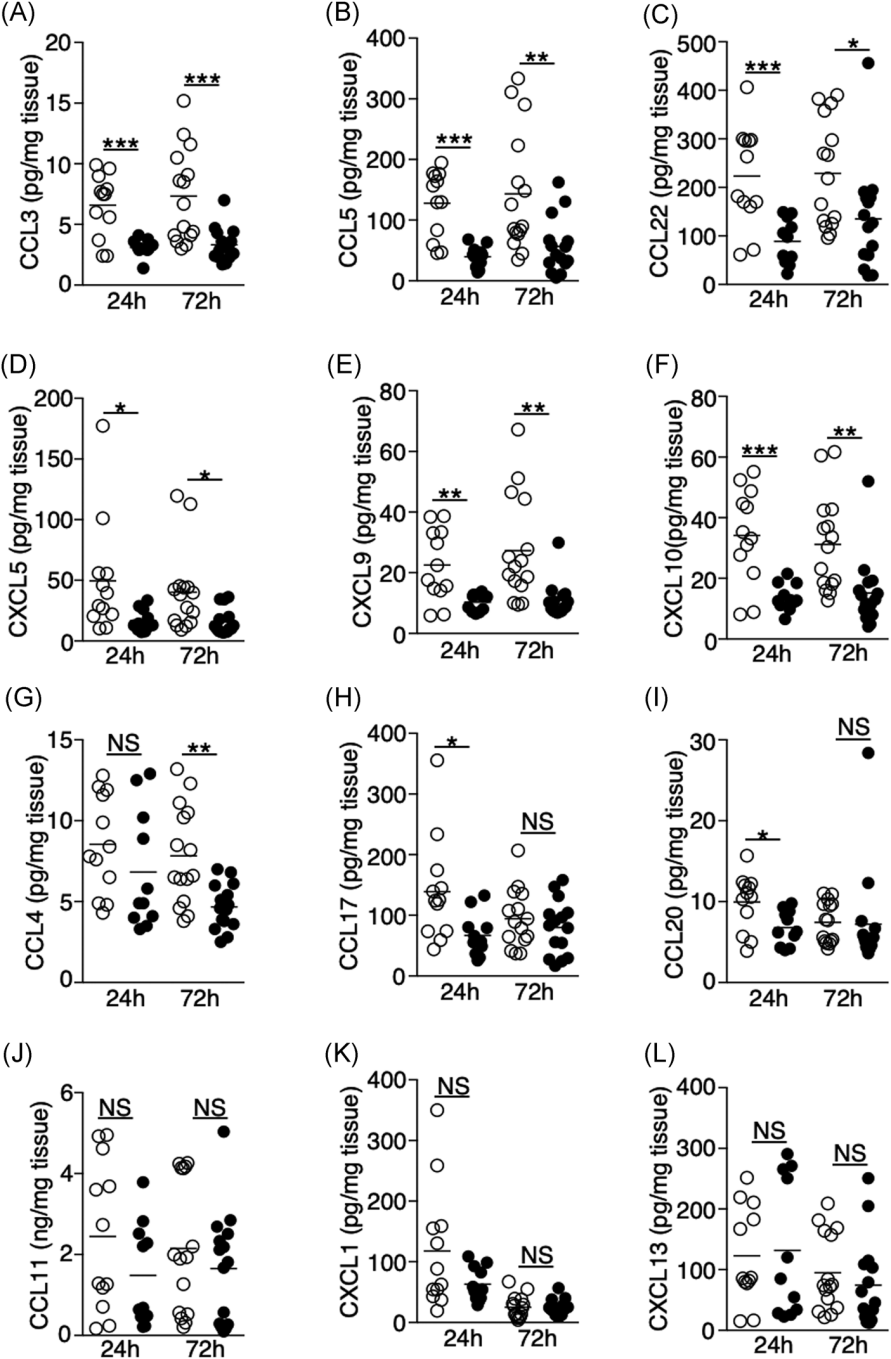
Ischemic stroke suppresses the production of multiple chemokines in the lungs. A‐L, Lung tissues were homogenized 24 and 72 hours following tMCAO (filled circle) or sham operation (open circle), level of CCL3 (A), CCL5 (B), CCL22 (C), CXCL5 (D), CXCL9 (E), CXCL10 (F), CCL4 (G), CCL17 (H), CCL20 (I), CCL11 (J), CXCL1 (K), CXCL13 (L) in the lungs of individual animals was determined by multiplex bead array. Data shown are combined results from three to four independent experiments with *n* = 12‐15 animals per group (sham 24 hours, tMCAO 24 hours, sham 72 hours, tMCAO 72 hours). *P < .05; **P < .01; ***P < .001. NS, not statistically different; tMCAO, transient middle cerebral artery occlusion

Chemokine and cytokine expression play a key role in regulating immune‐cell trafficking and function. Using multiplex bead arrays, we measured the expression of a total of 25 chemokines and cytokines in the lungs following ischemic stroke that are known to control inflammatory responses. Among the 13 chemokines we detected, the levels of CCL3, CCL5, CCL22, CXCL5, CXCL9, and CXCL10 were significantly reduced at both 24‐ and 72‐hour time points following tMCAO (Figure 8A‐F). The levels of CCL4, CCL17, and CCL20 were reduced only at one time point (Figure 8G‐I). The suppression of chemokine production was not caused by a general lung dysfunction as the levels of CCL11, CXCL1, and CXCL13 were comparable between the sham‐operated and tMCAO mice (Figure 8J‐L). Interestingly, CCL2 was the only chemokine measured that was increased in the lungs following tMCAO (Figure 4D). In contrast to the lung tissues, chemokine levels in the BALF were mostly unchanged following tMCAO, except for a reduction in CCL22 at both time points and CCL20 at 72 hours poststroke (Figure S3).

**Figure 9:**
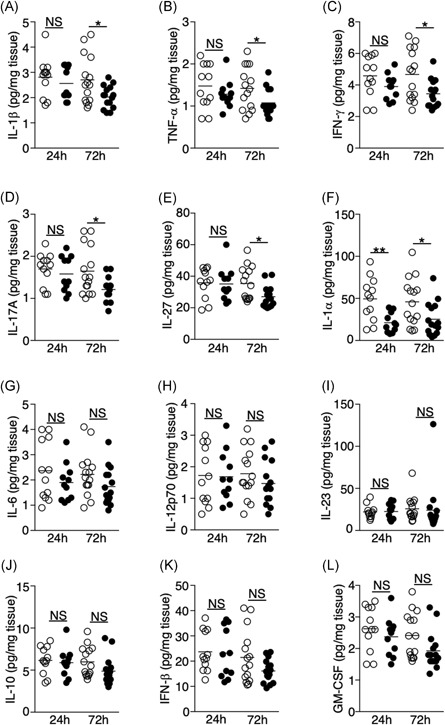
Ischemic stroke suppresses the production of multiple cytokines in the lung. A‐L, Lung tissues were homogenized 24 and 72 hours following tMCAO (filled circle) or sham operation (open circle), level of IL‐1β (A), TNF‐α (B), IFN‐γ (C), IL‐17A (D), IL‐27 (E), IL‐1α (F), IL‐6 (G), IL‐12p70 (H), IL‐23 (I), IL‐10 (J), IFN‐β (K), GM‐CSF (L) in the lungs of individual animals was determined by multiplex bead array. Data shown are combined results from three to four independent experiments with *n* = 12 to 15 animals per group (sham 24 hours, tMCAO 24 hours, sham 72 hours, tMCAO 72 hours). *P < .05; **P < .01. GM‐CSF, granulocyte‐macrophage colony‐stimulating factor; IFN, interferon; IL, interleukin; NS, not statistically different.different; tMCAO, transient middle cerebral artery occlusion; TNF‐α, tumour necrosis factor α

Proinflammatory cytokines such as IL‐1β, TNF‐α, and IL‐6 are critical for promoting bacterial clearance during lung infections.^19^, ^20^ IFN‐γ and IL‐17A are signature cytokines of Th1 and Th17 cells, both of which support innate‐cell activation and migration.^21^, ^22^ We found that the levels of IL‐1β, TNF‐α, IFN‐γ, IL‐17A, and IL‐27 were reduced 72 hours following ischemic stroke (Figure 9A‐E). Importantly, we found that IL‐1α was abundantly expressed in the lungs of the sham‐operated mice relative to other cytokines we measured (~50 pg/mg of tissue), but its level was significantly reduced at both 24‐ and 72‐hour time points postischemic stroke (Figure 9F). Levels of IL‐6, IL‐12p70, IL‐23, IL‐10, IFN‐β, and granulocyte‐macrophage colony‐stimulating factor (Figure 9G‐L) were unaltered following tMCAO. Significant changes in cytokine levels in BALF following tMCAO were not observed (Figure S4). Overall, these data suggest that ischemic stroke creates an immunosuppressive milieu in the lungs by decreasing the production of multiple proinflammatory chemokines and cytokines.

## 4 DISCUSSION

Our study demonstrated that ischemic stroke directly impacts the immune cell niche, and the availability of multiple chemokines and cytokines in the lungs, which was previously unexplored. These changes were unlikely due to the spontaneous bacterial infection that reported in other studies.^3^, ^15^ Consistent with our observations, recent studies have also shown that severe ischemic stroke in mice does not cause spontaneous bacterial infection.^23^ Using tMCAO model, Stanley et al^15^ showed that SAP is caused by the translocation and dissemination of commensal bacteria from the intestinal tracts to lungs, and SAP does not occur in mice housed in a germ‐free environment. These observations strongly suggest that the incidence of SAP is determined by the composition of gut microbiota, and this speculation is further supported by the fact that mice with different genetic backgrounds develop SAP with various severity.^11^ The incidence of SAP may also depend on factors other than genetic backgrounds such as the source of the animals and hygiene condition of the housing facility, which is commonly observed in many animal disease models.^24^ Importantly, it is still unclear whether gut bacterial translocation to the lungs is the cause of clinical SAP. While some SAP‐causative pathogens such as *Klebsiella pneumoniae* and *Escherichia coli* are commonly found in the gut microflora, *Staphylococcus aureus*, *Pseudomonas aeruginosa*, and *Streptococcus pneumoniae* are not.^25^ It is possible that clinical SAP results from a combination of nosocomial infection and bacterial translocation from the gut microflora. This possibility can be explored by comparing the composition of the gut microflora of patients with severe ischemic stroke that develop SAP with those patients that do not develop SAP.

Currently, it is still unclear how ischemic stroke‐mediated immune‐cell dysfunctions in remote tissues such as the spleen and the thymus lead to pneumonia. Our data suggested that alterations in the pulmonary immune cell niche following ischemic stroke may partly explain the lung‐specific immunodeficiency. A recent study has shown that neutrophils from the mouse bone marrow have an impaired chemotactic ability poststroke in an in vitro model.^9^ However, neutrophil infiltration to the lungs was observed in both spontaneous‐ or aspiration‐induced pneumonia following tMCAO.^3^, ^5^ We observed an increase in alveolar macrophages and the infiltration of neutrophils in the lungs following tMCAO, but their bacterial clearance capability remains to be elucidated. Oxidative burst and NETosis were reduced in neutrophils isolated from patients who experienced ischemic stroke.^26^ To connect these functional deficits with SAP, we are currently investigating the antibacterial activity of alveolar macrophages and neutrophils in the lungs following ischemic stroke using reporter mouse strains coupled with live‐imaging techniques. In addition, recruitment of monocytes during lung infection is critical for bacterial clearance.^27^ Clinically, reduction of HLA‐DR expression in monocytes is a prognostic marker of stroke‐induced immune suppression and SAP.^2^, ^28^, ^29^ Interestingly, we found that ischemic stroke increases the expression of the monocyte chemoattractant CCL2 in the lungs, yet monocyte infiltration into the lungs was not observed. Conversely, following ischemic stroke, we observed a massive infiltration of monocytes into the brain, which may account for temporal peripheral immune exhaustion following ischemic stroke.

This study is the first report showing a significant reduction of lymphocytes in the lungs following ischemic stroke that was not caused by the induction of cell death, but was coincided with the decreased production of multiple chemokines. The effect of ischemic stroke on regulating chemokine expression in the lungs is not known. Our data suggest that suppression of chemokine expression may “pre‐condition” the lungs to become vulnerable to bacterial infections. Particularly, CCL5 and CCL22 were abundantly expressed in the sham‐operated mice (>100 pg/mg of tissue), but their levels were significantly reduced following tMCAO. CCL5 is a potent chemoattractant of T cells to the site of inflammation, which may explain the reduction of these cells in the lungs following tMCAO. CCL5 antibody treatment in mice challenged with *S. pneumoniae* caused reductions in CD4^+^ and CD8^+^ T lymphocytes, resulting in dysregulation of a critical phase of the adaptive response. The loss of CCL5 ultimately led to a switch in bacteria from carrier state to lethal state of infection.^30^ CCL22 is known to promote Th2‐mediated immune responses such as airway hypersensitivity, atopic dermatitis, and eosinophilic pneumonia,^31^, ^32^, ^33^ but its role in bacterial infections is unclear. CCL22 is mainly produced by macrophages and DCs^33^, ^34^; reduction of CCL22 in the lungs indicates a possible functional impairment of these cells following ischemic stroke, which warrants further investigation.

There is an urgent need to develop effective immunotherapeutic strategies for SAP. Elucidating dysregulations in the pulmonary immune cell niche and the functions of the cells within this niche is a critical first step to achieve this goal. We demonstrated that ischemic stroke directly impacts pulmonary immunity. Restoration of the chemokine availability in the lungs may potentially prevent SAP in stroke patients.

## CONFLICT OF INTERESTS

The authors declare that there are no conflict of interests.

## AUTHOR CONTRIBUTIONS

EW and BF designed and performed experiments, analyzed data, and wrote the paper. KM, WZ, CA, and AA performed experiments and analyzed data. HH and XR performed experiments. JC analyzed data. EW supervised the project.

## Supporting information

Supporting information

Supporting information

Supporting information

Supporting information

Supporting information

Supporting information

Supporting information

## Data Availability

The data that support the findings of this study are available from the corresponding author upon reasonable request.

## References

[1] Benjamin EJ , Blaha MJ , Chiuve SE , et al. Heart disease and stroke statistics‐2017 update: a report from the American Heart Association. Circulation. 2017;135:e146‐e603. 28122885 10.1161/CIR.0000000000000485 PMC5408160

[2] Hoffmann S , Harms H , Ulm L , et al. Stroke‐induced immunodepression and dysphagia independently predict stroke‐associated pneumonia—The PREDICT study. J Cereb Blood Flow Metab. 2017;37:3671‐3682. 27733675 10.1177/0271678X16671964 PMC5718319

[3] Prass K , Meisel C , Hoflich C , et al. Stroke‐induced immunodeficiency promotes spontaneous bacterial infections and is mediated by sympathetic activation reversal by poststroke T helper cell type 1‐like immunostimulation. J Exp Med. 2003;198:725‐736. 12939340 10.1084/jem.20021098 PMC2194193

[4] Offner H , Subramanian S , Parker SM , Afentoulis ME , Vandenbark AA , Hurn PD . Experimental stroke induces massive, rapid activation of the peripheral immune system. J Cereb Blood Flow Metab. 2006;26:654‐665. 16121126 10.1038/sj.jcbfm.9600217

[5] Prass K , Braun JS , Dirnagl U , Meisel C , Meisel A . Stroke propagates bacterial aspiration to pneumonia in a model of cerebral ischemia. Stroke. 2006;37:2607‐2612. 16946159 10.1161/01.STR.0000240409.68739.2b

[6] Maier IL , Karch A , Mikolajczyk R , Bahr M , Liman J . Effect of beta‐blocker therapy on the risk of infections and death after acute stroke—a historical cohort study. PLOS One. 2015;10:e0116836. 25643360 10.1371/journal.pone.0116836 PMC4314079

[7] Chamorro A , Meisel A , Planas AM , Urra X , van de Beek D , Veltkamp R . The immunology of acute stroke. Nat Rev Neurol. 2012;8:401‐410. 22664787 10.1038/nrneurol.2012.98

[8] Wong CH , Jenne CN , Lee WY , Leger C , Kubes P . Functional innervation of hepatic iNKT cells is immunosuppressive following stroke. Science. 2011;334:101‐105. 21921158 10.1126/science.1210301

[9] Nicholls AJ , Wen SW , Hall P , Hickey MJ , Wong CHY . Activation of the sympathetic nervous system modulates neutrophil function. J Leukoc Biol. 2018;103:295‐309. 29345350 10.1002/JLB.3MA0517-194RR PMC6635748

[10] Clark WM , Lessov NS , Dixon MP , Eckenstein F . Monofilament intraluminal middle cerebral artery occlusion in the mouse. Neurol Res. 1997;19:641‐648. 9427967 10.1080/01616412.1997.11740874

[11] Schulte‐Herbruggen O , Klehmet J , Quarcoo D , Meisel C , Meisel A . Mouse strains differ in their susceptibility to poststroke infections. Neuroimmunomodulation. 2006;13:13‐18. 16612133 10.1159/000092109

[12] Liesz A , Dalpke A , Mracsko E , et al. DAMP signaling is a key pathway inducing immune modulation after brain injury. J Neurosci. 2015;35:583‐598. 25589753 10.1523/JNEUROSCI.2439-14.2015 PMC4293412

[13] Liesz A , Hagmann S , Zschoche C , et al. The spectrum of systemic immune alterations after murine focal ischemia: immunodepression versus immunomodulation. Stroke. 2009;40:2849‐2858. 19443795 10.1161/STROKEAHA.109.549618

[14] Chiu NL , Kaiser B , Nguyen YV , Welbourne S , Lall C , Cramer SC . The volume of the spleen and its correlates after acute stroke. J Stroke Cerebrovasc Dis. 2016;25:2958‐2961. 27615448 10.1016/j.jstrokecerebrovasdis.2016.08.012 PMC5154801

[15] Stanley D , Mason LJ , Mackin KE , et al. Translocation and dissemination of commensal bacteria in post‐stroke infection. Nat Med. 2016;22:1277‐1284. 27694934 10.1038/nm.4194

[16] Casson CN , Doerner JL , Copenhaver AM , et al. Neutrophils and Ly6Chi monocytes collaborate in generating an optimal cytokine response that protects against pulmonary Legionella pneumophila infection. PLOS Pathog. 2017;13:e1006309. 28384349 10.1371/journal.ppat.1006309 PMC5404877

[17] Craig A , Mai J , Cai S , Jeyaseelan S . Neutrophil recruitment to the lungs during bacterial pneumonia. Infect Immun. 2009;77:568‐575. 19015252 10.1128/IAI.00832-08 PMC2632043

[18] Xiong H , Keith JW , Samilo DW , Carter RA , Leiner IM , Pamer EG . Innate lymphocyte/Ly6C(hi) monocyte crosstalk promotes *Klebsiella Pneumoniae* clearance. Cell. 2016;165:679‐689. 27040495 10.1016/j.cell.2016.03.017 PMC4842125

[19] Zhang P , Summer WR , Bagby GJ , Nelson S . Innate immunity and pulmonary host defense. Immunol Rev. 2000;173:39‐51. 10719666 10.1034/j.1600-065x.2000.917306.x

[20] Strieter RM , Belperio JA , Keane MP . Host innate defenses in the lung: the role of cytokines. Curr Opin Infect Dis. 2003;16:193‐198. 12821807 10.1097/00001432-200306000-00002

[21] Chen K , Kolls JK . T cell‐mediated host immune defenses in the lung. Annu Rev Immunol. 2013;31:605‐633. 23516986 10.1146/annurev-immunol-032712-100019 PMC3912562

[22] Tsai HC , Velichko S , Hung LY , Wu R . IL‐17A and Th17 cells in lung inflammation: an update on the role of Th17 cell differentiation and IL‐17R signaling in host defense against infection. Clin Dev Immunol. 2013;2013:267971. 23956759 10.1155/2013/267971 PMC3730142

[23] Austin V , Ku JM , Miller AA , Vlahos R . Ischaemic stroke in mice induces lung inflammation but not acute lung injury. Sci Rep. 2019;9:3622. 30842652 10.1038/s41598-019-40392-1 PMC6403328

[24] Sundberg JP , Schofield PN . Living inside the box: environmental effects on mouse models of human disease. Dis Model Mech. 2018:11 10.1242/dmm.035360 PMC6215423 30194139

[25] Kishore AK , Vail A , Jeans AR , et al. Microbiological etiologies of pneumonia complicating stroke: a systematic review. Stroke. 2018;49:1602‐1609. 29915122 10.1161/STROKEAHA.117.020250

[26] Ruhnau J , Schulze K , Gaida B , et al. Stroke alters respiratory burst in neutrophils and monocytes. Stroke. 2014;45:794‐800. 24523038 10.1161/STROKEAHA.113.003342

[27] Goto Y , Hogg JC , Whalen B , Shih CH , Ishii H , Van Eeden SF . Monocyte recruitment into the lungs in pneumococcal pneumonia. Am J Respir Cell Mol Biol. 2004;30:620‐626. 14578212 10.1165/rcmb.2003-0312OC

[28] Urra X , Cervera A , Obach V , Climent N , Planas AM , Chamorro A . Monocytes are major players in the prognosis and risk of infection after acute stroke. Stroke. 2009;40:1262‐1268. 19164783 10.1161/STROKEAHA.108.532085

[29] Zhang DP , Yan FL , Xu HQ , Zhu YX , Yin Y , Lu HQ . A decrease of human leucocyte antigen‐DR expression on monocytes in peripheral blood predicts stroke‐associated infection in critically‐ill patients with acute stroke. Eur J Neurol. 2009;16:498‐505. 19187263 10.1111/j.1468-1331.2008.02512.x

[30] Palaniappan R , Singh S , Singh UP , et al. CCL5 modulates pneumococcal immunity and carriage. J Immunol. 2006;176:2346‐2356. 16455992 10.4049/jimmunol.176.4.2346

[31] Nureki S , Miyazaki E , Ando M , Kumamoto T , Tsuda T . CC chemokine receptor 4 ligand production by bronchoalveolar lavage fluid cells in cigarette‐smoke‐associated acute eosinophilic pneumonia. Clin Immunol. 2005;116:83‐93. 15925835 10.1016/j.clim.2005.03.001

[32] Katoh S , Fukushima K , Matsumoto N , et al. Accumulation of CCR4‐expressing CD4+ T cells and high concentration of its ligands (TARC and MDC) in bronchoalveolar lavage fluid of patients with eosinophilic pneumonia. Allergy. 2003;58:518‐523. 12757454 10.1034/j.1398-9995.2003.00149.x

[33] Yamashita U , Kuroda E . Regulation of macrophage‐derived chemokine (MDC, CCL22) production. Crit Rev Immunol. 2002;22:105‐114. 12433129

[34] Vulcano M , Albanesi C , Stoppacciaro A , et al. Dendritic cells as a major source of macrophage‐derived chemokine/CCL22 in vitro and in vivo. Eur J Immunol. 2001;31:812‐822. 11241286 10.1002/1521-4141(200103)31:3<812::aid-immu812>3.0.co;2-l

