# Supplementary material for: Ischemic stroke alters immune cell niche and chemokine profile in mice independent of spontaneous bacterial infection
Source: Immun Inflamm Dis. 2019 Nov 5;7(4):326–41. doi: 10.1002/iid3.277 (PMC6842816; doi:10.1002/iid3.277)

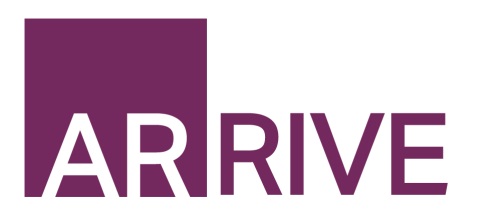


The ARRIVE Guidelines Checklist

Animal Research: Reporting In Vivo Experiments

Carol Kilkenny^1^, William J Browne^2^, Innes C Cuthill^3^, Michael Emerson^4^ and Douglas G Altman^5^

*^1^The National Centre for the Replacement, Refinement and Reduction of Animals in Research, London, UK, ^2^School of Veterinary Science, University of Bristol, Bristol, UK, ^3^School of Biological Sciences, University of Bristol, Bristol, UK, ^4^National Heart and Lung Institute, Imperial College London, UK, ^5^Centre for Statistics in Medicine, University of Oxford, Oxford, UK.*

|  | | ITEM | RECOMMENDATION | Section/ Paragraph |
| --- | --- | --- | --- | --- |
| 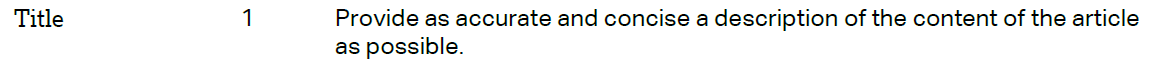 | | | Title |  |
| 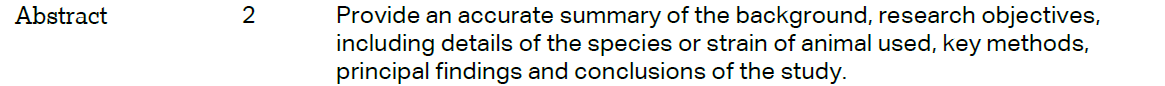 | | | Abstract |  |
| INTRODUCTION | | |  |  |
| 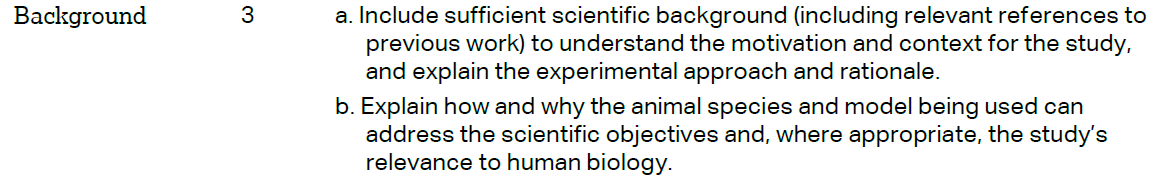 | | | Paragraphs  1-3  Paragraphs 2-3 |  |
| 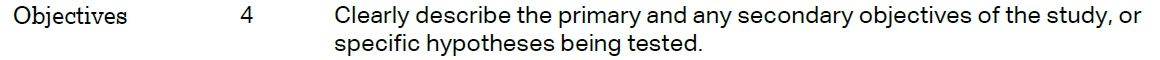 | | | Paragraph 2 |  |
| METHODS | | |  |  |
| 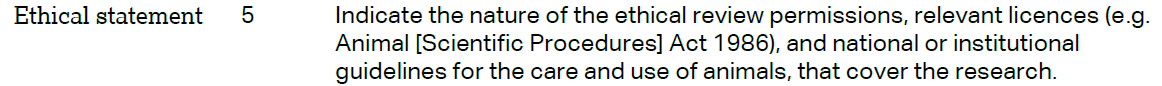 | | | Paragraph 1 |  |
| 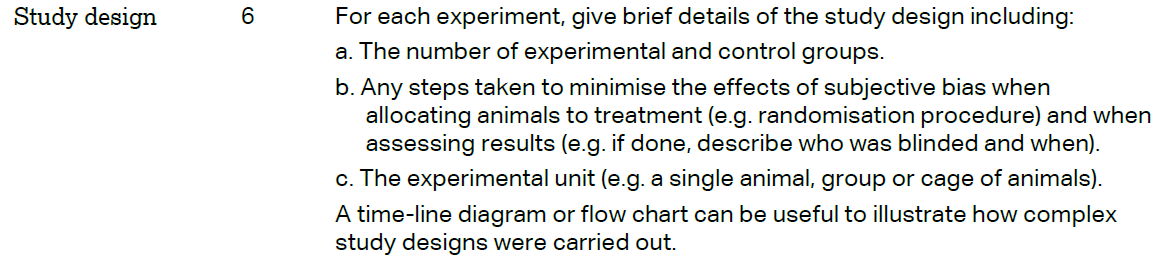 | | | Paragraph 2  Paragraph 2  Paragraph 2 |  |
| 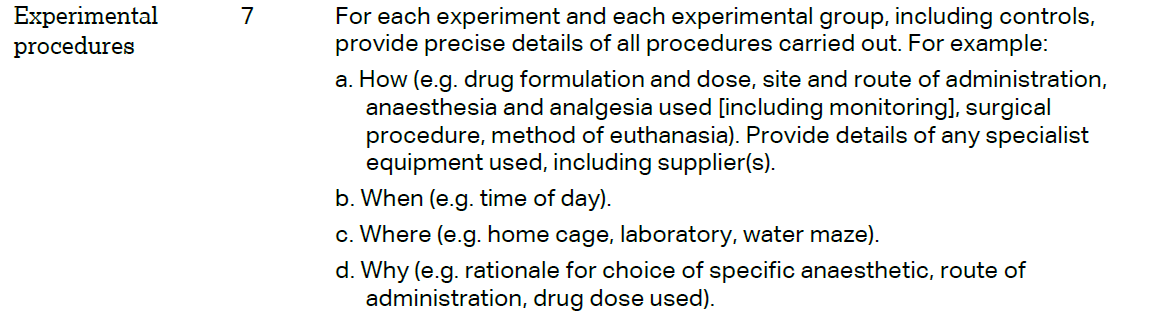 | | | Paragraph 2  Paragraph 2  Paragraph 2  Paragraph 2 |  |
| 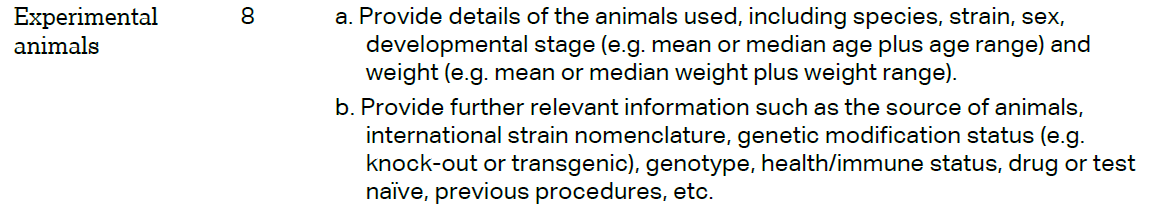 | | | Paragraph 1  Paragraph 1 |  |

The ARRIVE guidelines. Originally published in *PLoS Biology*, June 2010^1^

| 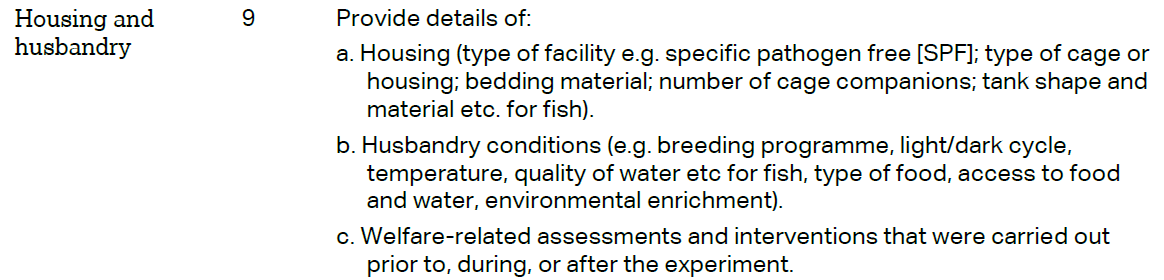 | Paragraph 1  Paragraph 1  Paragraphs 1-2 | |
| --- | --- | --- |
| 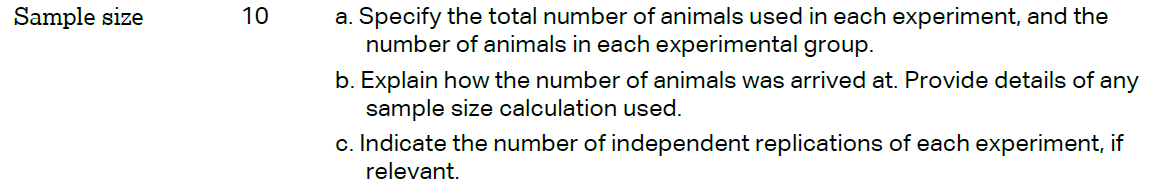 | Figure legends  Figure legends  Figure legends | |
| 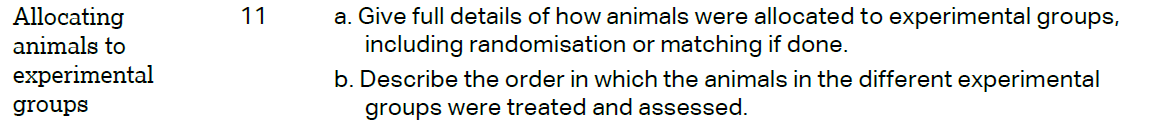 | Paragraph 2  Paragraph 3 | |
| 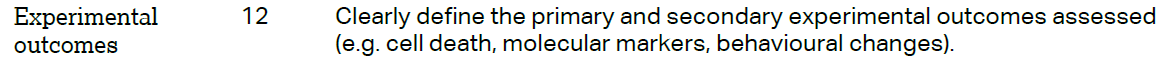 | Paragraph 3 | |
| 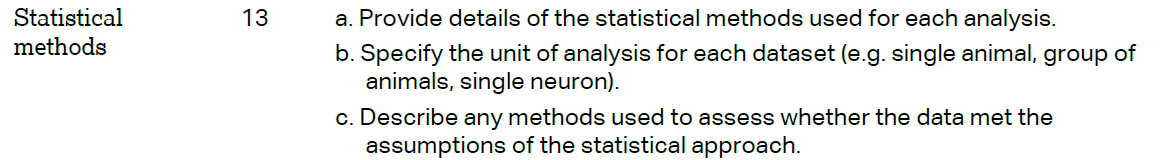 | Paragraph 14  Paragraph 14 | |
| RESULTS |  | |
| 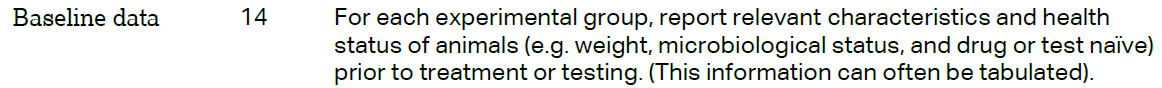 | Methods  Paragraph 1 | |
| 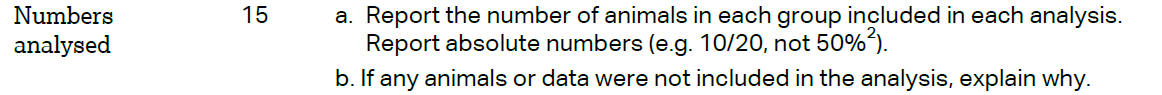 | Methods  Paragraph 2  Methods  Paragraph 2 | |
| 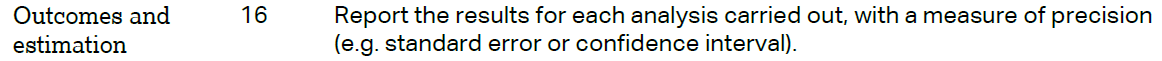 | Figure legends | |
| 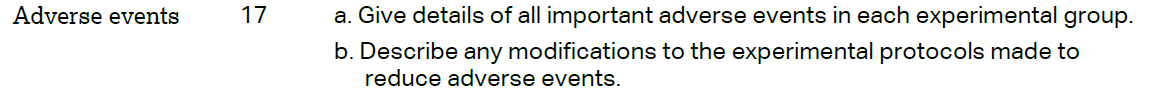 | Methods  Paragraph 2  Methods  Paragraph 2 | |
| DISCUSSION |  | |
| 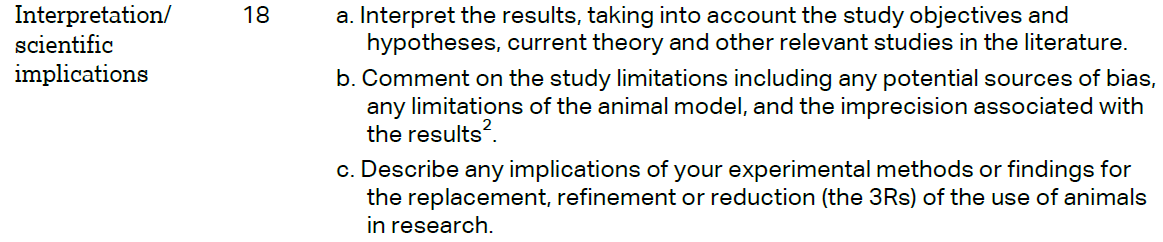 | Throughout  Paragraph 1  Methods Paragraph 2 | |
| 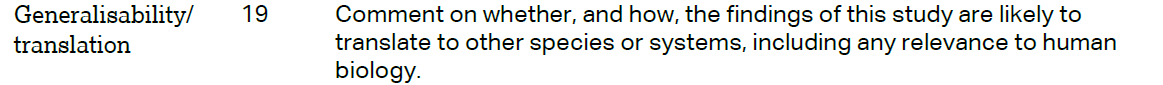 | Paragraph 4 | |
| 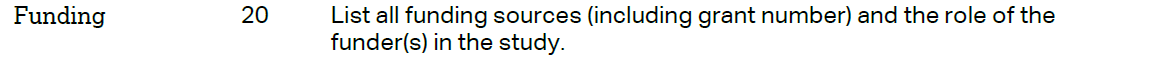 | | Submission system |


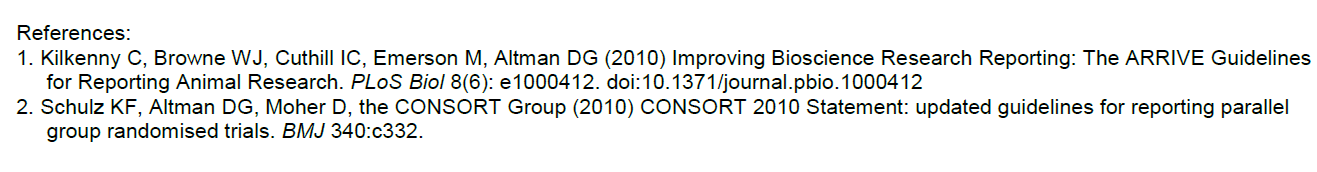

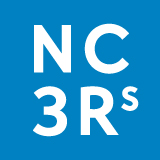

Supplement: Supplementary file 2 — Supporting information [file IID3-7-326-s002.docx]
