# Supplementary figures and images for: Ischemic stroke alters immune cell niche and chemokine profile in mice independent of spontaneous bacterial infection
Source: Immun Inflamm Dis. 2019 Nov 5;7(4):326–41. doi: 10.1002/iid3.277 (PMC6842816; doi:10.1002/iid3.277)

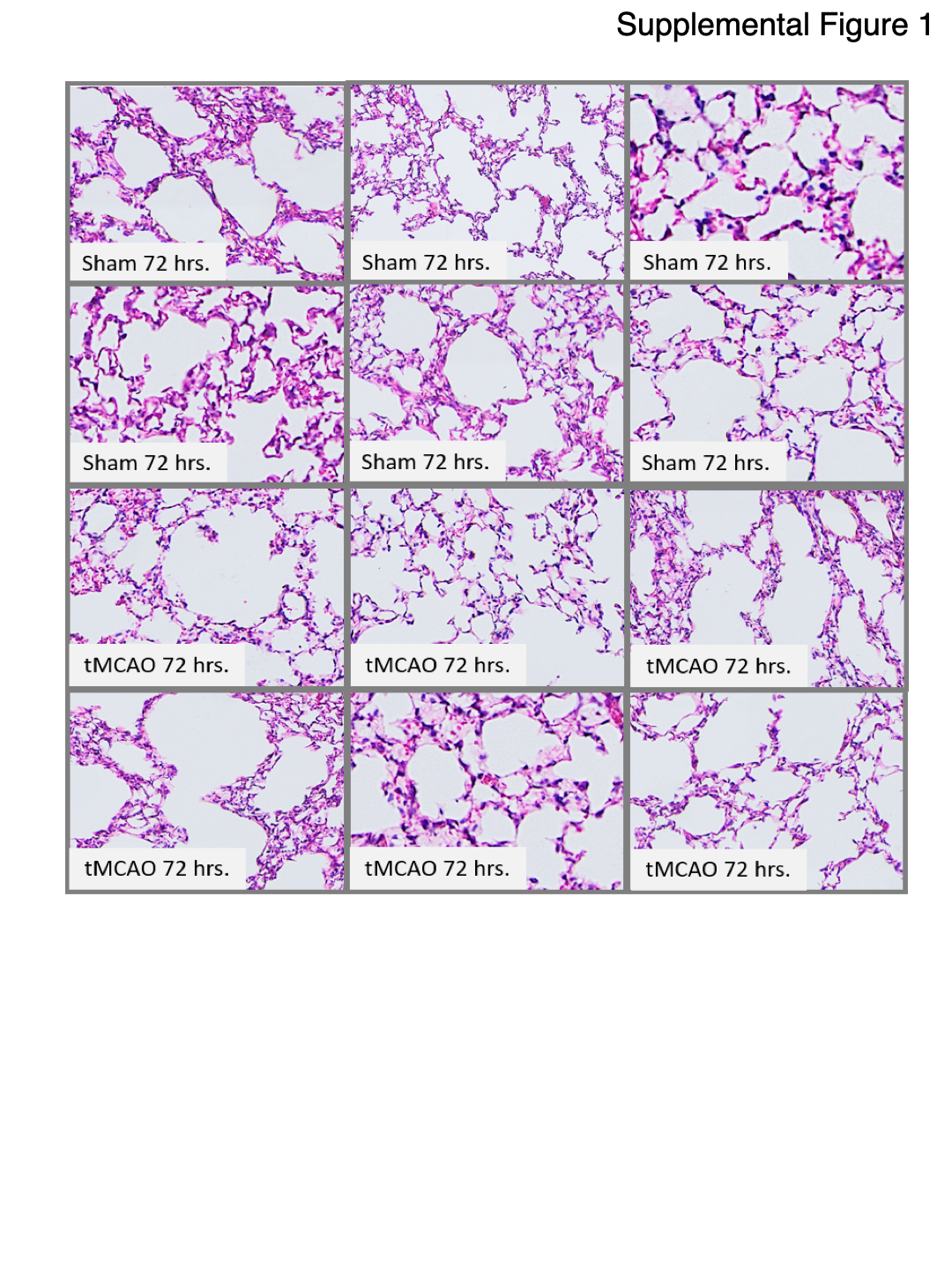

Supplement: Supplementary file 3 — Supporting information [file IID3-7-326-s003.tiff]

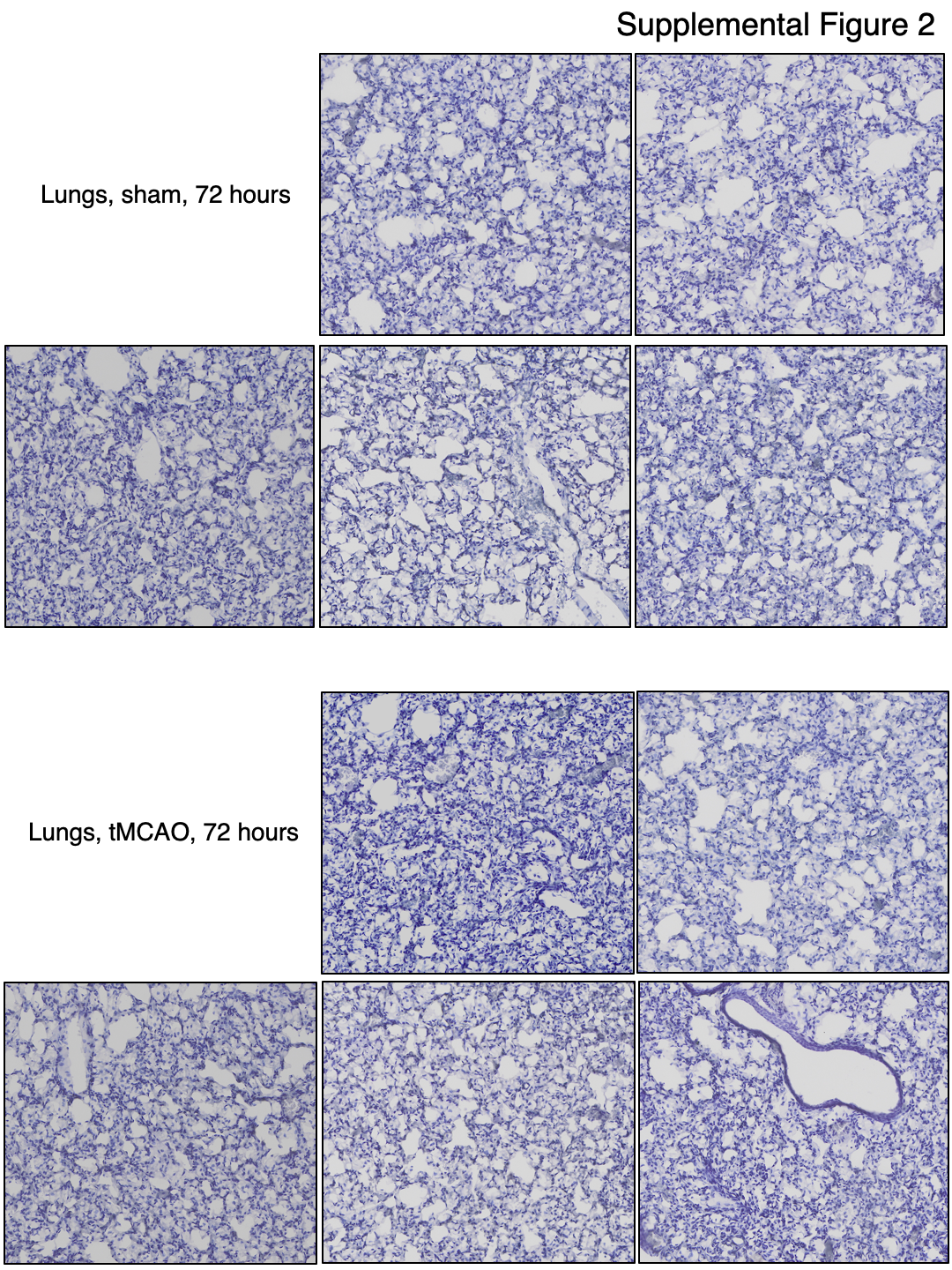

Supplement: Supplementary file 4 — Supporting information [file IID3-7-326-s004.tiff]

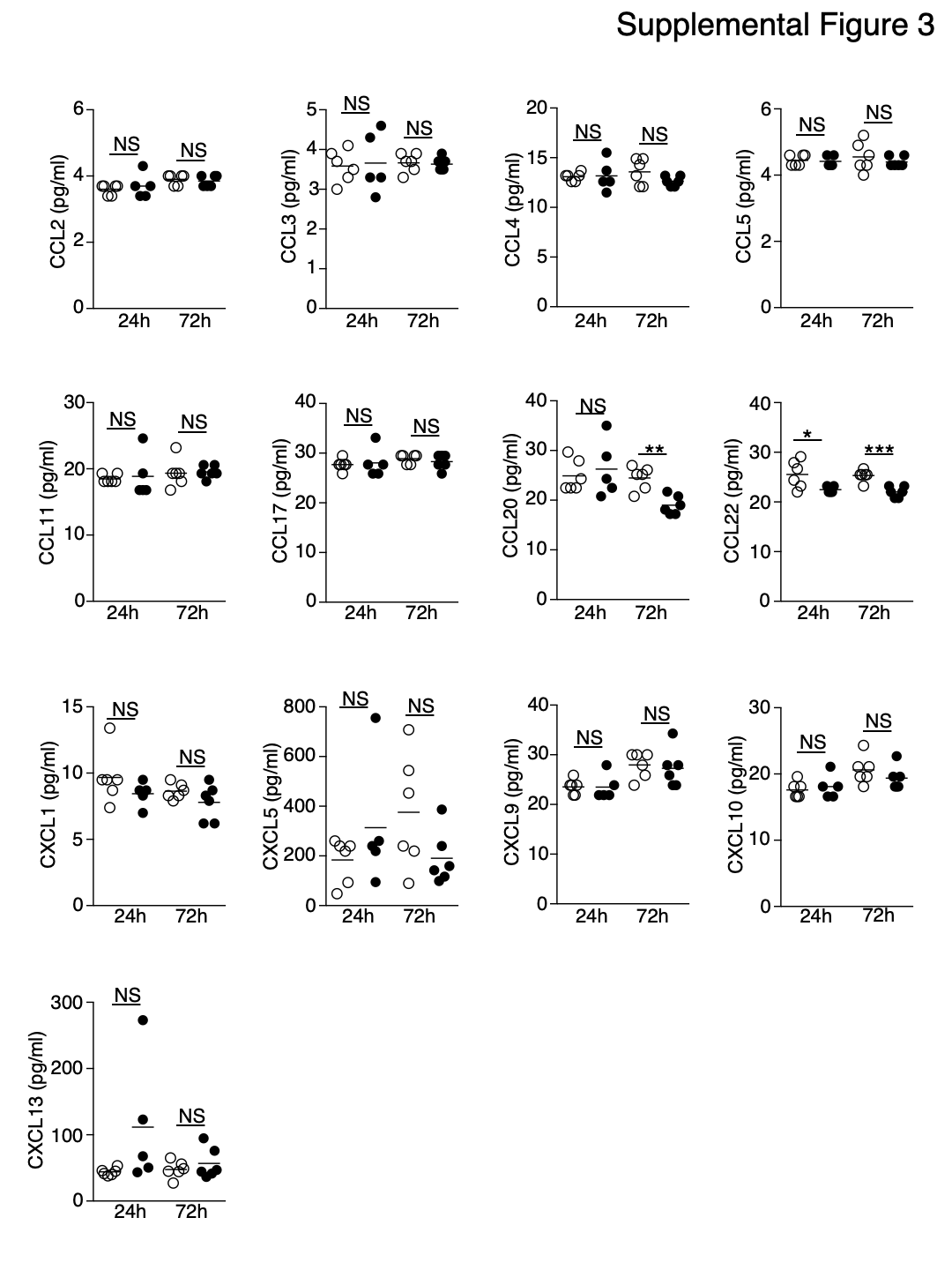

Supplement: Supplementary file 5 — Supporting information [file IID3-7-326-s005.tiff]

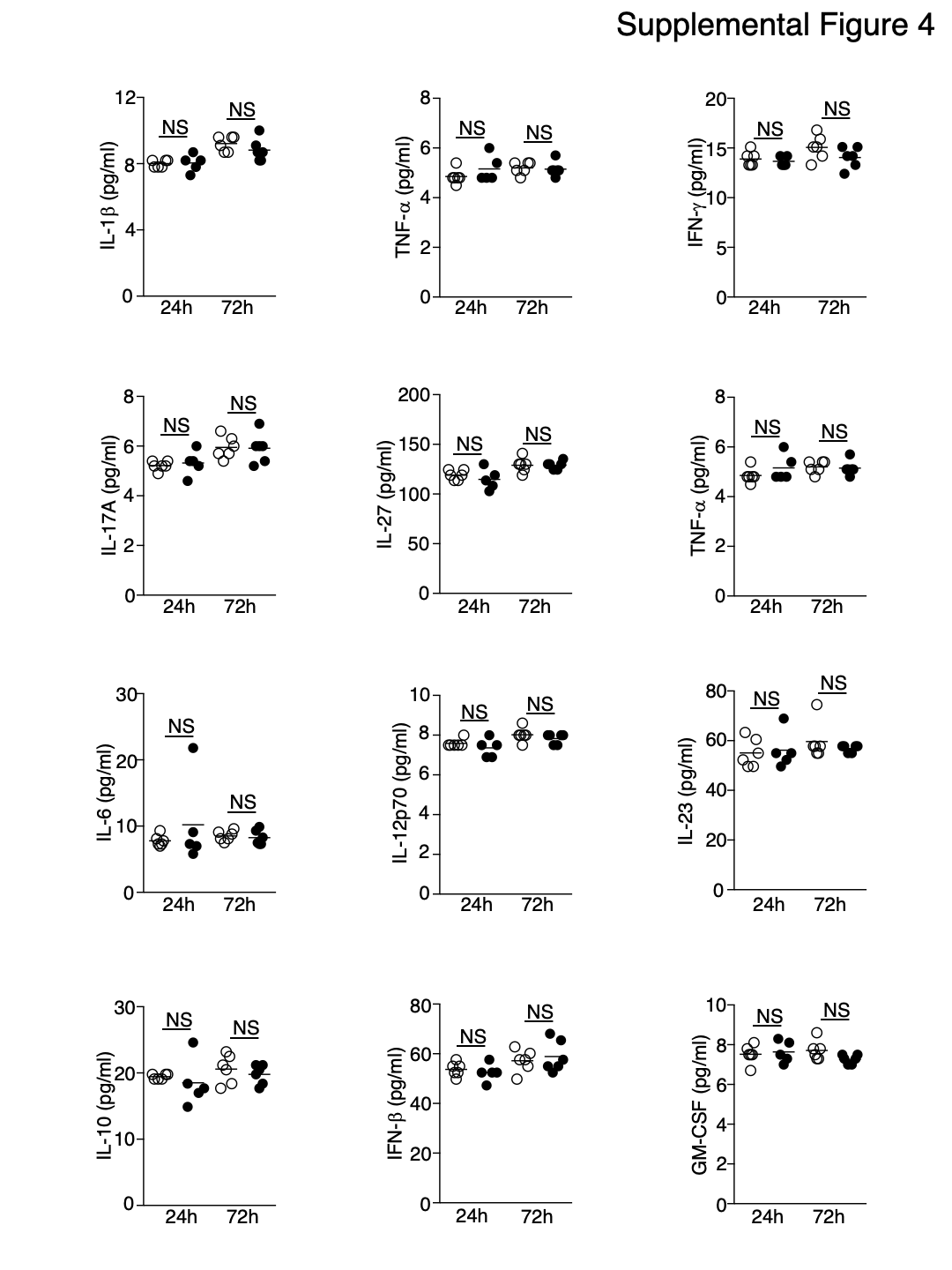

Supplement: Supplementary file 6 — Supporting information [file IID3-7-326-s006.tiff]
