# Supplementary material for: Ischemic stroke alters immune cell niche and chemokine profile in mice independent of spontaneous bacterial infection
Source: Immun Inflamm Dis. 2019 Nov 5;7(4):326–41. doi: 10.1002/iid3.277 (PMC6842816; doi:10.1002/iid3.277)
Supplement: Supplementary file 7 — Supporting information [file IID3-7-326-s007.docx]

**Figure S1.** Severe ischemic stroke in C57BL/6J mice does not cause spontaneous pneumonia. Lung pathology was examined 72 hours following tMCAO (bottom) or Sham operation (top). Shown are H and E images from all animals examined. Data shown are results from two independent experiments with n = 6 animals per group (sham 24h; tMCAO 24h; sham 72h; tMCAO 72h).

**Figure S2.** Ischemic stroke does not induce the activation of caspase 3 in the lungs. Lung tissues were dissected 72 hours following tMCAO or sham operation. The cleaved (activated) form of caspase 3 was measured by immunohistochemistry assay. Shown are images from all animals examined with n = 6 per group (sham 72h; tMCAO 72h)

**Figure S3**. Ischemic stroke reduces the level of CCL20 and CCL22 in the BALF. BALF was collected 24 and 72 hours following tMCAO (filled circle) or sham operation (open circle), level of chemokines described in Fig. 7 was determined. Data shown are combined results from two independent experiments with n = 5-6 animals per group (sham 24h; tMCAO 24h; sham 72h; tMCAO 72h). *, P < 0.05; **, P < 0.01; ***, P < 0.001. NS, not statistically different.

**Figure S4**. Ischemic stroke does not change the level of proinflammatory cytokines in the BALF. BALF was collected 24 and 72 hours following tMCAO (filled circle) or sham operation (open circle), level of cytokines described in Fig. 8 was determined. Data shown are combined results from two independent experiments with n = 5-6 animals per group (sham 24h; tMCAO 24h; sham 72h; tMCAO 72h). NS, not statistically different.

**Table S1.** Recovery of bacteria from the lungs 24 or 72 hours following tMCAO.
